# Preoperative treatment with mFOLFIRINOX or Gemcitabine/Nab-paclitaxel +/- isotoxic high-dose stereotactic body Radiation Therapy (iHD-SBRT) for borderline resectable pancreatic adenocarcinoma (the STEREOPAC trial): study protocol for a randomised comparative multicenter phase II trial

**DOI:** 10.1186/s12885-023-11327-x

**Published:** 2023-09-21

**Authors:** Christelle Bouchart, Julie Navez, Ivan Borbath, Karen Geboes, Timon Vandamme, Jean Closset, Luigi Moretti, Pieter Demetter, Marianne Paesmans, Jean-Luc Van Laethem

**Affiliations:** 1 Department of Radiation Oncology, Université Libre de Bruxelles (ULB), Hopital Universitaire de Bruxelles (H.U.B.), Institut Jules Bordet, Rue Meylenmeersch 90, 1070 Brussels, Belgium; 2 Department of Hepato-biliary-pancreatic surgery, Hopital Universitaire de Bruxelles H.U.B. - CUB Hopital Erasme, Université Libre de Bruxelles (ULB), Brussels, Belgium; 3 Department of Gastroenterology and Digestive Oncology, Cliniques Universitaires St-Luc, Brussels, Belgium; 4 Department of Gastroenterology, Digestive Oncology, UZ Gent, Corneel Heymanslaan 10, 9000 Gent, Belgium; 5 Department of Oncology, UZ Antwerpen, Drie Eikenstraat 655, 2650 Antwerpen, Belgium; 6 Department of Pathology, Université Libre de Bruxelles (ULB), Hopital Universitaire de Bruxelles (H.U.B.), Institut Jules Bordet, Rue Meylenmeersch 90, 1070 Brussels, Belgium; 7 Information Management Unit, Hopital Universitaire de Bruxelles (H.U.B.), Institut Jules Bordet, Rue Meylenmeersch 90, 1070 Brussels, Belgium; 8 Department of Gastroenterology, Hepatology and Digestive Oncology, Hopital Universitaire de Bruxelles H.U.B., Université Libre de Bruxelles (ULB), Route de Lennik 808, 1070 Brussels, Belgium

**Keywords:** Pancreatic adenocarcinoma, FOLFIRINOX, Stereotactic body radiation, Borderline resectable, Chemotherapy, Neoadjuvant therapy, Clinical trial

## Abstract

**Background:**

For patients with pancreatic ductal adenocarcinoma (PDAC), surgical resection remains the only potentially curative treatment. Surgery is generally followed by postoperative chemotherapy associated with improved survival, yet neoadjuvant therapy is a rapidly emerging concept requiring to be explored and validated in terms of treatment options and oncological outcomes. In this context, stereotactic body radiation (SBRT) appears feasible and can be safely integrated into a neoadjuvant chemotherapy regimen of modified FOLFIRINOX (mFFX) with promising benefits in terms of R0 resection, local control and survival. However, the optimal therapeutic sequence is still not known, especially for borderline resectable PDAC, and the role of adding SBRT to chemotherapy in the neoadjuvant setting needs to be evaluated in randomised controlled trials. The aim of the STEREOPAC trial is to assess the impact and efficacy of adding isotoxic high-dose SBRT (iHD-SBRT) to neoadjuvant mFFX or Gemcitabine/Nab-Paclitaxel (Gem/Nab-P) in patients with borderline resectable PDAC.

**Methods:**

This is a randomised comparative multicentre phase II trial, planning to enrol patients (n = 256) diagnosed with a borderline resectable biopsy-confirmed PDAC. Patients will receive 4 cycles of mFFX (or 6 doses of Gem/Nab-P). After full disease restaging, non-progressive patients will be randomised for receiving either 4 additional mFFX cycles (or 6 doses of Gem/Nab-P) (Arm A), or 2 mFFX cycles (or 3 doses of Gem/Nab-P) + iHD-SBRT (35 to 55 Gy in 5 fractions) + 2 mFFX cycles (or 3 doses of Gem/Nab-P) (Arm B). Then curative surgery will be performed followed by adjuvant chemotherapy according to patient’s condition. The co-primary endpoints are R0 resection and disease-free survival after the complete sequence strategy. The secondary endpoints include resection rate, overall survival, locoregional failure / distant metastasis free interval, pathologic complete response, toxicity, postoperative complications and quality of life assessment.

**Discussion:**

This trial will help define the best neoadjuvant treatment sequence for borderline resectable PDAC and aims to evaluate if a total neoadjuvant treatment integrating iHD-SBRT improves the patients’ oncological outcomes.

**Trial registration:**

The study was registered at ClinicalTrails.gov (NCT05083247) on October 19th, 2021, and in the Clinical Trials Information System (CTIS) EU CT database (2022-501181-22-01) on July 2022.

**Supplementary Information:**

The online version contains supplementary material available at 10.1186/s12885-023-11327-x.

## Introduction- background and rationale

Pancreatic ductal adenocarcinoma (PDAC) is a highly aggressive cancer with a poor prognosis mainly due to the high frequency of distant metastases or the locally advanced stage of the tumour excluding a surgical procedure from the outset [1]. In Belgium, with nearly 1,700 deaths a year, PDAC ranks fourth in the cancer mortality classification. According to some estimations, PDAC will reach the second place by 2030, especially in the western world [2]. This type of cancer has often few and late symptoms making the diagnosis more delayed. One-third of the patients are metastatic at diagnosis, less than 20% of the cases are immediately resectable and about 50% have a potentially resectable cancer called “borderline resectable” (BR) or locally advanced and unresectable (LAPC); the classification of resectability being determined according to the relationship of the tumour with the neighboring vascular structures [3]. All stages combined, the overall survival (OS) rate at 5 years is only 7%. Surgical resection is the only treatment modality offering a chance of cure. However, even operated patients have a poor prognosis with an OS at 5 years of 20%. Complete microscopic (R0) resection represents a requisite component of curative therapy for patients with PDAC [2, 4].

As a standard, surgery is usually followed by adjuvant therapy that significantly improves survival; this concept was proven by several trials (PRODIGE-24, CONKO-001 and ESPAC-4): usually 6 months of modified FOLFIRINOX (mFFX) regimen is administrated in fit patients (improved disease-free survival [DFS] by 40% from 12 to 18 months) and in more frail or older patients, gemcitabine monotherapy or in combination with capecitabine is given (improved DFS by 20%) [5–7]. However, only around 50% of the patients are able to complete full adjuvant therapy sequence [5–7]. Neoadjuvant therapy (NAT) is recommended in international guidelines, although this rapidly emerging concept still needs to be further explored and validated in BR PDAC [8–11]. Neoadjuvant regimen designed to both select patients for surgery and optimize surgical outcomes are needed. Yet the exact sequence and regimens remain to be determined, particularly regarding the potential additional benefit of a total neoadjuvant treatment (TNT) including stereotactic body radiation therapy (SBRT).

In recent randomised phase III trials, neoadjuvant chemotherapy (gemcitabine + S1) was shown to be efficacious in resectable tumours in Asian patients while in the PREOPANC trial, gemcitabine-based chemoradiation (CRT) reported, after a median follow-up of 59 months, a limited improvement in survival for resectable/BR PDAC (15.7 vs. 14.3 months, p = .025) [12, 13]. Recently, the results of the phase III CONKO-007 trial including 525 LAPC patients in which an induction mainly by mFFX was followed or not by CRT were presented. The primary endpoint, the R0 resection rate (RR), was not associated with a significant improvement (30 vs. 42% for CRT, p = .143) as well as for the OS and PFS, with the exception of the circumferential resection margin (CRM)-R0 RR (15 vs. 33% for CRT, p = .001) [14]. As reported in several prospective observational/phase II trials, preoperative mFFX prolonged by SBRT appears feasible and associated with promising outcomes in terms of R0 resection and prolonged survival [15, 16]. When compared with conventional long course CRT, SBRT offers several advantages such as the shorter duration of treatment (1 week vs. 4 to 6 weeks). Therefore, patients receiving SBRT can resume systemic therapy more quickly, reducing long interruptions of full-dose chemotherapy. Another advantage of SBRT is the improvement of local control with the possibility of delivering safely higher biologically effective doses (BED) to the tumour and the tumour/vessel interfaces (TVIs) [15, 16]. It is therefore an attractive option to propose both local control and a downstaging/shrinking of tumours with vascular contacts/invasion that preclude R0 resection. As the exact and best therapeutic sequence is not yet known, the role of adding SBRT to chemotherapy for BR PDAC requires validation in randomised trials. However, the type of pancreatic SBRT seems pivotal to yield the expected benefits as illustrated in the randomised phase II Alliance A021501 trial designed to compare the outcomes of BR patients treated with induction with FOLFIRINOX alone or followed by low-dose SBRT (33 Gy in 5 fractions with simultaneous integrated boost (SIB) up to 40 Gy at TVI or 25 Gy in 5 fractions) [17]. The results regarding the patients enrolled prior to closure for futility (70 patients enrolled in ARM A (FFX) and 56 in arm B (FFX + SBRT - underpowered) were recently published and the primary objective was not met (18-months OS rate: 67.9 vs. 47.3% in disfavor of the SBRT arm) [17]. In addition to several design shortcomings, reducing the delivered RT dose to or close to palliative dose range for safety purpose, as done in the Alliance trial, is not a solution as the maximal BED obtained (55 Gy) is well below the ablative doses sought with SBRT [15, 18]. It is essential to continue to improve the pancreatic SBRT technique, in particular by trying to deliver very high BED ≥ 70 Gy, as this seems correlated with improved OS and PFS on multivariate analysis [15, 19–22]. One safe method to achieve high-BED SBRT is to use an isotoxic dose prescription (IDP), based on organ at risk (OAR) tolerance levels and not on the tumour volume as usually done [16, 23]. Whilst protecting the OARs, the iHD-SBRT technique permits to reach the highest achievable dose level to increase the local tumour control probability and to safely obtain a BED10 ≥ 70 Gy [16, 24]. We recently reported feasibility and preliminary efficacy data of the TNT sequence combining preoperative FFX x 6 cycles (or Gemcitabine/Nab-Paclitaxel [Gem/Nab-P] in case of intolerance or no response) prolonged by iHD-SBRT (SIB up to 53 Gy at the TVI in 5 fractions) with promising results [16].

The present study proposes now to evaluate the impact and efficacy of incorporating iHD-SBRT to preoperative neoadjuvant mFFX or Gem/Nab-P in patients with BR PDAC in a TNT sequence. We hypothesize that this full sequence strategy of pre-operative treatment is safe and feasible and will improve both the surgical management (R0 resection) and the prognosis of PDAC patients as evaluated by the DFS, as co-primary end-points.

## Method

The SPIRIT reporting guidelines were used for this protocol and the completed SPIRIT checklist is available in Supplementary Table 1 [25].

### Trial design

**Fig. 1:**
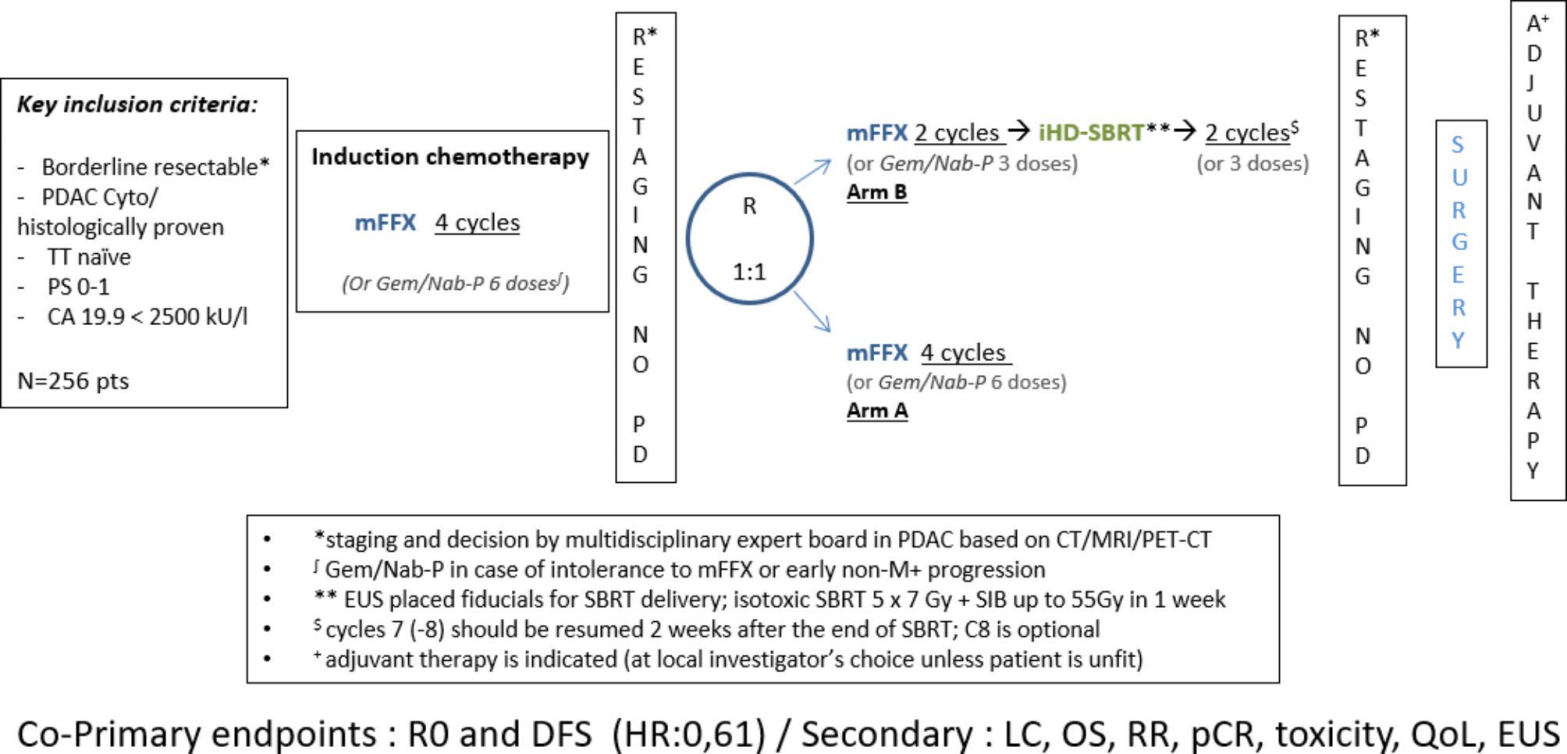
Overview of treatment sequences of the trial

This study is a multicenter randomised open-label comparative phase II trial. Patients who have been diagnosed with borderline resectable PDAC (according to the NCCN definition [8]) will be enrolled. Patients are initially planned to be recruited at 10 centers in Belgium with expertise in pancreatic surgery, including collaborative centers within the Belgian healthcare convention. Patients will receive 4 cycles of mFFX (or 6 doses of Gem/Nab-P) (Fig. 1). After full disease restaging, non-progressive patients will be randomised for receiving either 4 mFFX cycles (or 6 doses of Gem/Nab-P) (Arm A), or 2 mFFX cycles (or 6 doses of Gem/Nab-P) + iHD-SBRT followed by 2 additional mFFX cycles (or 6 doses of Gem/Nab-P) (Arm B). After full disease restaging, a curative surgery will be performed consisting of oncological pancreatectomy, followed by adjuvant chemotherapy according to patient’s condition. In case of contraindication to mFFX, early intolerance or loco-regional progression, Gem/Nab-P regimen can be chosen for 12 doses (6 doses followed by restaging, then either 6 doses in arm A, or 3 doses / iHD-SBRT / 3 doses in arm B), before surgery. Surgery is planned to be performed ideally 6–8 weeks (maximum 10 weeks) after iHD-SBRT delivery for Arm B and ideally 3–4 weeks (maximum 6 weeks) after the last cycle of chemotherapy for Arm A.

### Randomization and minimization of bias

The randomisation will be performed in subjects after receiving the first 4 cycles doses of FFX (or 6 doses Gem/Nab-P), showing a manageable tolerance and no progressive disease at restaging. Subjects will be centrally and automatically randomised electronically by the RedCap® system.

To minimize the imbalance in the distribution of treatment numbers within the levels of each individual prognostic factor, a minimization technique will be used for random treatment allocation stratifying by centers and CA 19.9 levels.

### Objectives and end-points

The co-primary objectives are to compare both the R0 resection rate (a R0 resection is considered if the distance between the inked margins and the tumour cells is > 1 mm, with a central pathology review) and the disease-free survival (DFS) in arm A (‘standard’-chemotherapy) to arm B (‘experimental’- chemotherapy + iHD-SBRT) in an intention-to-treat analysis. DFS is defined as time from randomization to the first documentation of one of the following events: radiological disease progression (defined according to the RECIST criteria version 1.1 [26]), discovery of hepatic metastasis or peritoneal carcinomatosis during surgery, recurrent disease following curative surgery or death due to any cause. Secondary objectives include: resection rate, OS (by intention-to-treat), locoregional failure free interval (LFFI), distant metastases free interval (DMFI), complete feasibility of the therapeutic sequence, pathologic complete response rate (pCR), toxicity (early and late), postoperative complications rate, quality of life (QoL) assessment at different timepoints and technical/quality success rate of endoscopic ultrasound (EUS)-delivered fiducials. LFFI is defined as the time interval between the randomisation and the date of locoregional failure, and DMFI as the period of time without distant metastasis after randomization. The complete feasibility of the therapeutic sequence is defined as the proportion of patient who performed completely the allocated neoadjuvant sequence until surgery. pCR is defined as the proportion of patients in whom a complete or a major response (< 10% of residual tumour cells) is confirmed by histopathologic review of the surgical specimen. The toxicity is assessed per Common Terminology Criteria for Adverse Events (CTCAE) version 5.0 [27]. Postoperative complications are graded according to the Clavien-Dindo classification and the Comprehensive Complication Index (CCI, calculated on https://www.cci-calculator.com/cciCalculator) and quality of life is assessed per EORTC QLQ-C30 version 3.0, QLQ-PAN26 questionnaires and PHQ-9 scale [28–32]. The technical success of EUS-delivered fiducials is defined as at least one marker presumed to be inside at the end of the EUS procedure. The quality success is defined as a score equal or higher than 6/12 points based on the quality score defined in Figueiredo et al. 2021 [33].

### Inclusion and exclusion criteria

Patients will be first included in the study on the basis of the following criteria.

*Inclusion criteria*.

- Cytologic or histologic proof of adenocarcinoma of the pancreatic head or uncinated process or body or tail. Diagnosis should be verified by local pathologist.
- TNM stage: T1-4N0-2M0.
- Confirmation of *clinical and radiographic stage* as borderline resectable determined by review of a high-quality diagnostic multisliced triphasic computed tomography (CT) and/or magnetic resonance (MRI) with contrast by a multidisciplinary board composed by a dedicated oncological pancreatic surgeon, radiologist and oncologist; according to the NCCN criteria (v1.2022 [8]).
- Age ≥ 18 years old.
- No prior chemotherapy or radiation for pancreatic cancer unless the neoadjuvant regimen as described.
- Eastern Cooperative Oncology Group (ECOG) performance status 0 or 1.
- No grade ≥ 2 neuropathies.
- Laboratory parameters as follows: absolute neutrophil count (ANC) ≥ 1,500/mm³, platelet count ≥ 100,000/mm³, hemoglobin ≥ 9 g/dL, creatinine ≤ 1.5 x upper limit of normal (ULN) or estimated glomerular filtration rate (GFR) > 45 mL/min, bilirubin ≤ 1.5 x ULN (including after adequate biliary stenting with metal stent), aspartate aminotransferase (AST) / alanine aminotransferase (ALT) ≤ 2.5x ULN, CA 19.9 < 2500 kU/l (baseline and absence of cholestasis).

*Exclusion criteria*.

- Evidence of extrapancreatic disease on diagnostic imaging or laparoscopy, including distal nodal involvement beyond the peripancreatic tissue and/or distant metastases.
- Unresectable disease as defined by the NCCN criteria, i.e. > 180° arterial encasement (superior mesenteric and coeliac arteries), unreconstructible or fully thrombosed venous invasion and/or occlusion of a long segment [8].
- CA 19.9 > 2500 kU/l (baseline and in absence of cholestasis).
- Contraindication to surgery.
- Contraindications to receive mFFX or Gem/Nab-P.
- History of radiotherapy of the upper abdomen.
- Prior treatment with oxaliplatin, irinotecan, fluoruouracil or capecitabin.
- Age < 18 years old.
- Major surgery within 4 weeks of study entry.
- Pre-existing uncontrolled disease including, but not limited to: active infection, symptomatic congestive heart failure, unstable angina, social/psychiatric disorder that would limit adherence to treatment and understanding of the informed consent form.
- Other concurrent anticancer therapies.
- Existence of another active neoplasia other than basal cell carcinoma of the skin, carcinoma in situ or non-metastatic prostate cancer. Patients with an history of neoplasia must be in remission for more than 5 years to be included in the protocol.
- Pregnant or breastfeeding women; for women of childbearing potential only, a negative pregnancy test taken < 7 days prior to enrollment is required. Use of reliable contraception for at least 1 month prior to treatment is mandatory.
- Chronic concomitant treatment with strong inhibitors of the cytochrome p450, family 3, subfamily a, polypeptide 4 gene (CYP3A4) is not authorized in this study; patients on potent CYP3A4 inhibitors must discontinue medication for 14 days prior to study enrollment.

*Specific and additional exclusion criteria before randomization and after initial chemotherapy*.

- Progressive disease (RECIST or PET-CT, including non-locoregional nodal involvement and increase of CA 19.9 by 20%) after receiving 4 cycles of FFX (or 6 doses Gem/Nab-P), including after a shift of chemotherapy in case of early progression/intolerance.
- CA19.9 level > 1000 kU/l after 4 cycles of FFX (or 6 doses Gem/Nab-p).
- Presence of unmanageable toxicity during the first part of neoadjuvant chemotherapy (first 4 cycles or 6 doses of FFX or Gem/Nab-P, respectively.
- Pancreatic tumour > 7.0 cm in greatest axial dimension at the time of randomization.
- Massive invasion of the stomach or intestines and/or direct intestinal invasion of the mucosae visible at ultrasound endoscopy.
- Active gastric or duodenal ulcer disease at the time of randomization; tolerated in case of antecedent without active ulcer (confirmation by endoscopy before iHD-SBRT).

### Therapeutic schedules (Fig. 2)

**Fig. 2:**
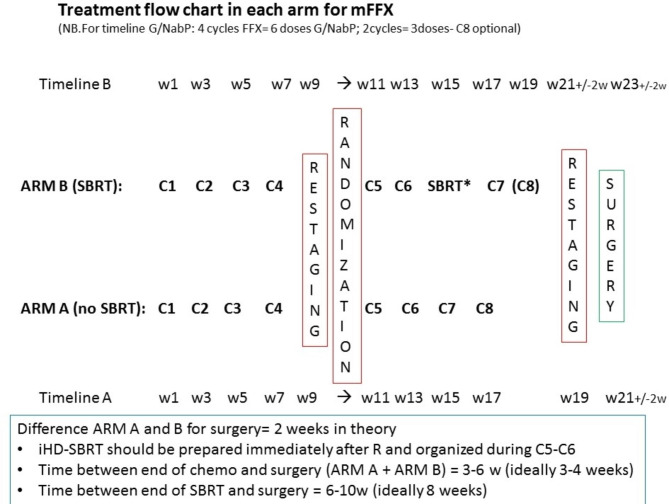
Treatment flow chart in each arm for FOLFIRINOX (NB. For timeline Gem/Nab-P: 4 cycles FFX = 6 doses Gem/Nab-P; 2 cycles FFX = 3 doses Gem/Nab-P; C8 optional)

#### Neoadjuvant chemotherapy regimens

Chemotherapy will be administrated at oncologist’s discretion and includes mFFX or Gem/Nab-P. mFFX consists of oxaliplatin (85 mg/m²), irinotecan (165–180 mg/m²), folinic acid (400 mg/m²) and 5-fluorouracil (2000–2400 mg/m²/46 h) regimen for 6 cycles every 2 weeks. Gem/Nab-P consists of gemcitabine (1000 mg/m² weekly, 3 weeks/4) and nab-paclitaxel (125 mg/m², weekly 3 weeks/4).

#### Isotoxic high-dose SBRT

For patients randomized to the iHD-SBRT arm, the iHD-SBRT will be started minimum 1 week and maximum 4 weeks after the end of chemotherapy (6th cycle of FFX or 9th dose of Gem/NabP). Fiducial markers placement (pre-loaded Cook ECHO-TIP F 22G™, Polymarks™ [RT-Idea] or LumiCoil™ platinum fiducial markers [Boston Scientific] charged 19G needle) in the pancreatic tumour under endo-ultrasonography (EUS) guidance is required for all patients receiving SBRT minimum 5 days before the SBRT simulation, as previously described, with the exception of MR-Linac treatments [16, 32]. A minimum of 4-h fasting is required before CT simulation with adequate immobilization device and respiratory motion assessment. iHD-SBRT will be delivered in five consecutive daily fractions according to an IDP [16]. The target dose will be individually maximized to the highest achievable level with simultaneous integrated boost to the tumour and TVIs up to D_max(0.5 cc)_ < 55 Gy and until at least one of the mandatory dose constraint levels for OARs is reached. The following OARs dose constraints must be respected: for planning organ at risk volume (PRV) stomach, duodenum, colon and small bowel, D_max (0.5 cc)_ < 40 Gy; for PRV spinal cord, D_max (0.5 cc)_ < 25 Gy; for kidneys, D_mean_<14 Gy and V_12Gy_ < 25%; and for liver; D_mean_<15 Gy and D_700cc_ < 21 Gy. A RT quality assurance (QART) manual has been developed and is available to all the participating centres. Pre- and post-treatment QA technical review (including dummy run) and audits will be conducted in order to ensure the protocol compliance and the adequate iHD-SBRT delivery.

#### Surgery

Intra-abdominal exploration with or without pancreatectomy (with minimum multiple biopsies if complete surgery not feasible) ideally within 6 to 8 weeks and maximum 10 weeks after iHD-SBRT completion (Arm B) and ideally 3–4 weeks (maximum 6 weeks) after the last cycle of chemotherapy for Arm A. The final decision to perform a pancreatectomy or not is left to the final judgment of the specialist surgeon at the time of surgery. Surgery will be performed only in expert centers as defined by the Belgian healthcare convention. Procedures will be performed by laparotomy or laparoscopy according to surgeon’s preference, under general anesthesia, and include either pancreatoduodenectomy, distal pancreatectomy or total pancreatectomy. Vascular resection and reconstruction will be performed in case of suspicion of vascular involvement.

#### Adjuvant chemotherapy

Adjuvant chemotherapy is indicated for a 4 months period unless patient’s condition after surgery precludes it (regimen left to investigator’s choice) and should be started within 3 months after surgery.

### Assessment and follow-up

All patients included in the study will be followed up through monitoring visits every 3 months for the first 2 years, then every 6 months for the next 3 years. They will perform clinical examination with evaluation of late toxicity, laboratory tests including CA 19 − 9 levels and CT of the chest and the abdomen. In case of recurrence, additional treatment will be proposed at the discretion of the local oncologist. The Table 1 resumes the schedule of enrolment, interventions and assessments of the STEREOPAC trial.

**Table 1:** Flow chart of the schedule of enrolment, interventions and assessments of the STEREOPAC trial

	Screening − 28 - D1	Pre-treatment− 14 − D1 Treatment period	Treatment period											1st FU = 15 (+/-7) days after surgery (EOT)	FU every 3 mo. for the first 2 y, then every 6 months for the next 3 y
			D1 (cycle 1)	Before C2 and C4	Before cycle 3	15 days after C4	RESTAGING & RANDOMISATION ^i^	After rando, before C5	Before C6	Before SBRT (Arm B) Radiotherapy	Before C7-C8	Before surgery	Surgery		
Informed consent	**X**														
Demographic data	**X**														
Eligibility criteria	**X** ^**e**^	**X** ^**e**^				**X** ^**g**^									
Medical history	**X**														
Echo-endoscopy	**X**									**X** ^**d**^					
Residual tumoural block or 10 unstained slides of tumour block (FFPE)	**X** ^**a**^												**X** ^**a**^		
Plasma samples	**X** ^**a**^											**X** ^**a**^			**X** ^**a**^
Concomitant medication		**X**	**X**	**X**	**X**	**X**		**X**	**X**	**X**	**X**	**X**		**X**	**X** ^**b**^
Physical exam	**X** ^**e**^	**X** ^**e**^	**X** ^**e**^	**X**	**X**	**X**		**X**	**X**	**X**	**X**	**X**		**X**	**X** ^**b**^
Urine dipstick		**X**													
Thoraco-abdominal CT and/or abdominal MRI	**X**					**X**				**X** ^**d,h**^		**X**			**X** ^**b,f**^
Restaging						**X**						**X**			
FDG-PET (optional)		**X** ^**c**^			**X**							**X**			
CA19-9 and CEA		**X** ^**e**^	**X** ^**e**^			**X**		**X**				**X**		**X**	**X** ^**b**^
ECOG PS	**X** ^**e**^	**X** ^**e**^	**X** ^**e**^	**X**	**X**	**X**		**X**	**X**	**X**	**X**	**X**		**X**	**X** ^**b**^
Cardiac assessment (ECG)	**X** ^**e**^	**X** ^**e**^										**X**			
Lab assessment	**X** ^**e**^	**X** ^**e**^	**X** ^**e**^	**X**	**X**	**X**		**X**	**X**	**X**	**X**	**X**		**X**	**X** ^**b**^
(S)AE reporting (CTCAE)			**Continuous**												
Pregnancy test		**X**													
QoL scales	**X** ^**e**^	**X** ^**e**^	**X** ^**e**^			**X**						**X**			**X** ^**l**^
Prescription of proton-pump inhibitor	**X** ^**j**^							**X** ^**d**^	**X** ^**d**^	**X** ^**d**^	**X** ^**d**^	**X** ^**d**^			
Decision of adjuvant chemotherapy															**X** ^**k**^
Survival and cause of death															**X** ^**b**^

a: only for patients participating in the ancillary translational study of the trial. After surgery, samples will be collected at the 3 months FU then every 6 months for the 2 first years

b: every 3 months for 2 years after the “First Follow-Up Visit (15 days after surgery (EOT)). Thereafter every 6 months during 3 years

c: assessment only needed if older than 28 days before start treatment

d: only for patients randomized in arm B with radiotherapy

e: If screening/eligibility check and pre-treatment visits are on the same day as D1, no double study related tasks need to be performed

f: no CT/MRI to be done anymore after progression

g: Additional exclusion criteria before randomization to be checked

h: CT / MRI simulation in RT treatment position, at Follow-up visit at 3 months (+/-1 month) after surgery

i:. Subjects whose tumor is staged as resectable at the initial restaging will continue the planned treatment cycles until planned surgery

FU: Follow-up; EOT: end of treatment

j: only in case of active gastrointestinal ulcer at the diagnosis echo-endoscopy

k: at Follow-up visit at 3 months (+/-1 month) after surgery

l: At the clinical FU 3 months after surgery, then every 6 months for the 2 first years

### Sample size and statistics

The co-primary endpoints of the study are both R0 resection and DFS after randomisation in the two treatment groups. R0 resection rate is expected to be 40% in the control arm and the difference worth to detect was an absolute increase of 20% (from 40 to 60%) [34]. Based on the current literature, a median DFS of 11 months post-randomisation was chosen for statistical design, and is expected to be prolonged up to 18 months (which translates into HR:0.61 with the assumption of exponential distributions) [35]. To adjust for two primary comparisons, a two sided α-error of 0.025 and β-error of 0.20, yielding a power of 80%, we assessed sample size separately for both objectives and chose the largest sample size.

For R0 resection, we need a sample size of 230 evaluable patients. Taking into account a 10% inevaluability rate, 256 patients need to be included to reach the objective related to R0 resection rate.

For DFS, 160 events will be required; with 5 patients included/ month, this number of events could be reached with the randomisation of 206 patients (both arms). An interim analysis (IA) for futility will be performed after 80 events and is expected to be feasible after the inclusion of 160 patients. Bêta spending Lan-Demets functions will be used to control for multiplicity. The study could be stopped for futility if, at the IA, the HR estimate is in the interval [0.90; 1.12]).

The study duration is then estimated to be 111 months (51 months for accrual + up to 5 years of follow-up).

Safety will be assessed after the first 20 patients having received the TNT sequence and surgery. Safety analysis will then be planned every six months. The final decision to terminate the trial in case the criteria for stopping rules and futility are encountered will be done by the sponsor of the study.

The statistical analysis of efficacy will consist for DFS on HR testing and for R0 resection on the testing of the difference between R0 resection rates. Analysis will also make use of Kaplan-Meier analysis of DFS, OS and duration of response, in the intention-to-treat population. Multivariable analysis of DFS and OS by means of Cox proportional hazards.

### Quality assessment of the trial

Chemotherapy will be administered as standard regimens for both mFFX or Gem/Nab-P.

iHD-SBRT will be delivered under a prespecified protocol supervised by the coordinating RT (CB) including QART as followed: each RT site must be credentialed for pancreatic SBRT prior to enrollment (facility questionnaire and dummy run procedure), the first 3 cases treated by iHD-SBRT must be centrally reviewed and approved before the beginning of the treatment (contouring and RT plan) and all the following RT treatments will be centrally reviewed retrospectively.

Surgery will be performed by expert pancreatic surgeons in dedicated expert centers as it is currently requested in Belgium by the healthcare program. The coordinating surgeon (JN) will chair an expert surgical committee that will review the procedures and potential complications after TNT.

Pathological evaluation and full margins assessment will be centrally reviewed by a committee under the supervision of the coordinating pancreatic pathologist (PD) and reported according to the recommendations from the Pancreatobiliary Pathology Society [36].

### Monitoring

Throughout the trial, an external independent expert committee will be responsible for monitoring and reviewing the data and make recommendations. Audits will periodically be performed in each participating center in order to randomly check compliance with the protocol, compliance with in- and exclusion criteria, proper implementation, conduct of Informed Consent procedures, data verification (i.e. crosscheck data in RedCap® with patient dossier and vice versa), and adequate reporting of serious adverse events (SAEs). Adverse events are graded using the CTCAE version 5.0 [27]. Suspected Unexpected Serious Adverse Reactions (SUSARs) are reported to the Competent Authority and Ethics Committee according to national regulation.

### Study termination

The termination of the study is at the discretion of the sponsor in any of the following events:

- Medical or ethical reasons affecting the continued execution of the study.
- Difficulties in recruiting patients.
- Occurrence of adverse events unknown to date as to their nature, severity and duration, or unexpected incidence/severity of known adverse events.

Study safety data will be reviewed by the sponsor and investigators on an ongoing basis to ensure that continuation of the study is appropriate.

### Ethical considerations

The study has been approved by the central Ethical Committee through the Clinical Trials Information System (CTIS) procedure (approval obtained on November 9th, 2022; reference number: 2022-501181-22-01). It has been registered at the ClinicalTrails.gov database (NCT05083247) on October 19th, 2021. The protocol has been designed according to the principles of Good Clinical Practice of the International Conference on Harmonization and of the Declaration of Helsinki. All patients will provide a written informed consent before starting treatment. An additional written inform consent will be obtain for the patients participating in the ancillary translational study for the collection of biological specimens. Subjects are free to discontinue the study at any time without giving their reason(s). Model of inform consent is available in Supplementary Material (Additional File 1 & 2). All the data collected during the study will be coded and the data entry will be done through the RedCap© software.

## Discussion

Neoadjuvant treatment is a rapidly emerging concept that still needs further investigation and validation in the treatment of BR PDAC. While several chemotherapeutic regimens and radiotherapeutic techniques have recently proven their relative efficacy, the best sequence and regimens remain to be determined [15, 37, 38]. The present study proposes to evaluate the impact of incorporating iHD-SBRT to preoperative neoadjuvant mFFX or Gem/Nab-P in patients with borderline resectable PDAC in a novel neoadjuvant sequence.

The mFFX chemotherapy regimen has been reported as safe, feasible, and active prior to surgery in phase II trials [39, 40]. It has also become one of the first choices in first line for patients with metastatic PDAC with a good performance status without severe co-morbidity since the PRODIGE4/ACCORD11 study. Several other studies confirmed its superiority over gemcitabine alone at the cost of increased toxicity [41–43]. FFX regimen achieves a median survival of only 11 months in metastatic patients [41]. FFX became the preferred chemotherapy regimen of many centers in the neoadjuvant approach. Recent retrospective studies and a meta-analysis showed that this neoadjuvant regimen seems to be the most effective with significantly better secondary resection rate and OS than other chemotherapy regimen [37, 38, 44]. Modifications of FFX regimen (attenuated doses of 5-fluorouracile and/or irinotecan) are widely used to improve tolerability, and decrease adverse events. Both retrospective and recent prospective trials showed comparable efficacy in metastatic disease and in LAPC [45]. FOLFIRINOX regimen in an adapted dose is also currently the standard of care in the adjuvant setting with a significant improvement in DFS and OS [5].

The combination of gemcitabine and nab-paclitaxel was also reported as efficient in the metastatic setting [46–48]; it was also recently reported in the neoadjuvant setting in phase II trials [33, 47]; the SWOG trial reported similar data using either FFX or Gem/Nab-P regimens in a randomised phase II with no true benefit on DFS after surgery but a significant rate of major pathological response [32]. It can be therefore considered as a valuable alternative to FFX in case of contraindications.

SBRT uses the latest technological advances developed over the past two decades in the field of radiotherapy, both in dose delivery and image-guided radiotherapy. SBRT allows the delivery of high ablative equivalent biological doses in few sessions (1 to 5) at the level of the gross tumour volume (GTV) in a highly conformational way, which makes it possible to reduce the dose and therefore the toxicity to nearby OAR [15]. The SBRT technique can be easily integrated into a neoadjuvant approach and the results of available Phase I/II and retrospective studies are promising, showing very high rates of R0 resection for BR and LAPC patients (84–97.5%) [15]. Therefore, and despite the lack of level I evidence, leading international (radio)oncological societies have already listed SBRT as a treatment option for localized PDAC in their guidelines [8–11]. In order to preliminary evaluate the feasibility and safety of iHD-SBRT in a total neoadjuvant sequence for the treatment of localized pancreatic adenocarcinoma, we have already performed a prospective analysis in 39 consecutive BR (18/39) and LAPC (16/39) patients [15]. Our results showed that iHD-SBRT displays an excellent toxicity profile, also for potentially high-risk patients with radiological direct gastrointestinal tumoral invasion at diagnosis, and can be easily integrated in a total neoadjuvant strategy. The oncological outcomes were also promising including: median OS and DFS of 24.5 and 15.6 months, respectively, a 1-year local control of 74.1%, a median locoregional DFS not reached and late/early gastrointestinal toxicity < 5% [15]. Given the lack of comparative evidence, we also retrospectively compared the outcomes of PDAC patients treated with iHD-SBRT (n = 41) or conventional CRT (n = 41) in the same tertiary cancer center [24]. The mOS was in favour of the iHD-SBRT group (22.5 vs. 15.9 months, p < .001) even after multivariate analysis (HR 0.39 [CI95% 0.18–0.83], p = .014) [24]. The iHD-SBRT group also dispayed significantly better median PFS and 1y-LC (median PFS: 16.7 vs. 11.5 months, p = .011; 1-yLC: 75.8 vs. 39.3%, p = .004) [24]. All of these results further support the implementation of SBRT treatments for PDAC patients and highlight the need for well-designed studies in this area, particularly investigating the benefit of adding optimally delivered iHD-SBRT in a total neoadjuvant setting that emerges now in other cancers [49].

## Conclusion

The purpose of this prospective randomised phase II is to evaluate the impact and efficacy of incorporating isotoxic high-dose SBRT into a total neoadjuvant treatment sequence including active chemotherapy for borderline resectable pancreatic adenocarcinoma. Therefore, this trial will help to define the best standard neoadjuvant treatment sequence.

### Trial status

Protocol version n°: 1.2 Date: October 18, 2022.

Date of beginning of recruitment: March 15, 2023.

Approximate date of complete accrual: 1/1/2028.

The STEREOPAC trial is a multicenter randomized phase II trial, conducted in 10 initial centers that provide multidisciplinary treatment for pancreatic cancer throughout Belgium. At the time of submission of this paper, all initial centers were activated. The first patient was included on March 24, 2023.

### Electronic supplementary material

Below is the link to the electronic supplementary material.

Supplementary Material 1

Supplementary Material 2

Supplementary Material 3

## Data Availability

Not applicable.

## Declarations

### Committees

Steering Committee.

From ULB: C. Bouchart, J. Navez, L. Moretti, M. Paesmans, P. Demetter, N. D’haene, J. Closset, JL. Van Laethem.

Form other Belgian universities: I. Borbath, C. Hubert, G. Van Ooteghem, T. Vandamme, G. Roeyen, K. Geboes, F. Berrevoets.

Patients’ group overseeing the trial : PISARO group (Institut Jules Bordet).

Statistician: Mrs Marianne Paesmans, MSc, Institut Jules Bordet - Data Centre.

Data management team: BGDO trial office: Ine De Bruyne (i.debruyne@bgdo.org) and Lina Dewever (lina.dewever@bgdo.org).

Administrative coordination: Mia Persoons, Clinical Research Unit, Department of Gastroenterology and Digestive Oncology, CUB Hôpital Erasme (mia.persoons@ hubruxelles.be ).

Study coordinators at sponsor’s site: Axelle Ghilain (Axelle.ghilain@ hubruxelles.be) and/or Camilla Previtali (Camilla.previtali@ hubruxelles.be), Clinical Research Unit, Department of Gastroenterology and Digestive Oncology, CUB Hôpital Erasme.

## Abbreviations

iHD-SBRT: Isotoxic High Dose Stereotaxic Body Radiation Therapy
PDAC: Pancreatic ductal adenocarcinoma
R0: Complete microscopic resection
SBRT: Stereotaxic Body Radiation Therapy
mFFX: Modified FOFIRINOX
Gem/Nab-P: Gemcitabine/Nab-Paclitaxel
TNT: Total neoadjuvant treatment
BR: Borderline resectable
LAPC: Locally advanced pancreatic cancer
OS: Overall survival
FFX: FOFIRINOX
DFS: Disease-free survival
NAT: Neoadjuvant therapy
RT: Radiation therapy
BED: Biologically effective dose
TVI: Tumour/vessel interface
SIB: Simultaneous integrated boost
IDP: Isotoxic dose prescription
OAR: Organ at risk
mDFS: Median disease-free survival
RECIST: Response evaluation criteria in solid tumours
LFFI: Locoregional failure free interval
DMFI: Distant metastases free interval
pCR: Pathologic complete response rate
QoL: Quality of life
EUS: Endoscopic ultrasound
CTCAE: Common Terminology Criteria for Adverse Events
EORTC: European Organisation for Research and Treatment of Cancer
CT: Computed tomography
MRI: Magnetic resonance imaging
ECOG: Eastern Cooperative Oncology Group
ANC: Absolute neutrophil count
ULN: Upper limit of normal
AST: Aspartate aminotransferase
ALT: Alanine aminotransferase
GFR: Glomerular filtration rate
NCCN: National Comprehensive Cancer Network
PET: Positron emission tomography
PRV: Planning organ at risk volume
QA: Quality assurance
HR: Hazard ratio
IA: Interim analysis
QART: Quality assurance of radiation therapy
SAE: Serious adverse events
SUSAR: Suspected Unexpected Serious Adverse Reactions
