# Supplementary material for: Preoperative treatment with mFOLFIRINOX or Gemcitabine/Nab-paclitaxel +/- isotoxic high-dose stereotactic body Radiation Therapy (iHD-SBRT) for borderline resectable pancreatic adenocarcinoma (the STEREOPAC trial): study protocol for a randomised comparative multicenter phase II trial
Source: BMC Cancer. 2023 Sep 21;23:891. doi: 10.1186/s12885-023-11327-x (PMC10512504; doi:10.1186/s12885-023-11327-x)
Supplement: Supplementary file 1 — Supplementary Material 1 [file 12885_2023_11327_MOESM1_ESM.pdf]

Main clinical study

STEREOPAC

Preoperative treatment with mFOLFIRINOX (or Gem-Nab-P) +/- isotoxic high-dose Stereotactic Body Radiation Therapy (iHD-SBRT) for borderline resectable pancreatic adenocarcinoma: a randomised comparative multicentre phase II study

EU number: 2022-501181-22-01

Trial number : NCT05083247 - STEREOPAC

Sponsor: CUB Hôpital Erasme (ULB), Route de Lennik 808, B- 1070 Brussels

Coordinating center: CUB Hôpital Erasme (ULB), Route de Lennik 808, B-1070 Brussels

Coordinating Investigator for Belgium: Prof. Dr. JL Van Laethem, CUB Hôpital Erasme (ULB), Department of Gastroenterology, Hepato-Pancreatology and Digestive Oncology, jl.vanlaethem@erasme.ulb.ac.be, Phone: +32 2 555 3714

Ethics Committee that issued the opinion on the study: CTIS & FAGG approval

Participating centers:

Prof. Dr. J-L Van Laethem (CUB Hôpital Erasme (ULB), Route de Lennik 808, B- 1070 Brussels)
Prof. Dr. I. Borbath (Cliniques Universitaires Saint-Luc, Avenue Hippocrate 10, 1200 Brussels)
Dr. T. Vandamme (Universitair ziekenhuis Antwerpen, Wilrijkstraat 10, 2650 Edegem)
Prof. Dr. K. Geboes (UZ Gent, Corneel Heymans 10, 9000 Gent)
Dr. G Houbiers (Clinique CHC MontLégia, Bd Patience et Beaujonc 9, 4000 Luik)
Dr. P. Vergauwe (AZ Groeningen, President Kennedylaan 4, 8500 Kortrijk)
Dr. Christelle Bouchart (Institut Jules Bordet, Rue Meylemeersch 90, 1070 Anderlecht)
Dr. Francesco Puleo (CHIREC Boulevard du Triomphe 201, 1160 Brussels)
Dr. A. Dermine (Pôle Hospitalier Jolimont, Rue Ferrer 159, 7100 Haine Saint Paul)
Dr. S. Dingenen (Centre Hospitalier Universitaire et Psychiatrique de Mons Borinage (CHUPMB), Boulevard Kennedy 2, 7000 Mons)

Document Revision History:

Version No.	Version Date	Revision description
1.0	27 June 2022	Initial version

Who can I contact in case of questions?

Name	Function	In case of	Contact details
Surname, first name	Principal Investigator of the site	Information, problems or concerns	Phone N°:<br>E-mail:
	The trial staff	Information, problems or concerns	Phone N°:
	Emergency contact	Emergency	Phone N°:
	Patient rights ombudsman	Concerns relating to your rights as a participant in a trial	Phone N°:<br>E-mail:
Ethias Assurance<br>Rue des Croisiers, 24<br>4000 Liège	Insurance Company of the sponsor	In case of disagreement or complaint on a damage claim	Policy N°: 45.415.046<br>Phone N°: 04/220.31.11
	Data protection officer of the site	Questions relating to the confidentiality of your data	Phone N°:<br>E-mail:
	Belgian Data Protection Authority	Complaints relating to the confidentiality of your data	E-mail: contact@apd-gba.be

TABLE OF CONTENTS

Document Revision History: ... 1

Who can I contact in case of questions? ... 2

THE TRIAL AT A GLANCE ... 5

DEAR PATIENT, ... 5

CHAPTER I – DESCRIPTION OF THE TRIAL AND YOUR RIGHTS WHEN PARTICIPATING ... 8

1. Why are we doing this trial ... 8

2. Why am I being asked to take part? ... 8

3. Do I have to take part in a trial? ... 10

4. What will happen during the trial? ... 10

5. Will I benefit from the trial? ... 16

6. What are the possible risks and discomforts of taking part? ... 16

6.1. What are the possible side effects of the treatment? ... 16

6.2. What are the possible risks or discomforts of the examinations during the trial? ... 19

6.3. Can I take other medicines during the trial? ... 20

6.4. Will my participation to the trial have an impact on my daily activities? ... 20

6.5. Can my partner or I get pregnant or can I breastfeed during the trial? ... 20

7. What If something goes wrong within the trial? ... 21

8. What if other treatment options or new information on the IMP become available during the course of the trial? ... 21

9. Can my participation in the trial end prematurely? ... 21

9.1. You decide to withdraw your consent ... 22

9.2. The investigator decides to end your trial participation ... 22

9.3. Other entities may interrupt or end the trial ... 23

10. Which treatment will I get after my participation in the trial? ... 23

11. Will my participation in the trial involve extra costs for me? ... 23

11.1. Examinations and treatments paid by the sponsor ... 23

11.2. Other expenses ... 23

12. Which data are collected about me during the trial and what will happen with them? 24

12.1. Which data are collected and processed during the trial? ... 24

12.2. How will the investigator treat my personal data? ... 24

12.3. What will happen to information about me collected during the trial? ... 24

12.4. How will my data be handled? ... 25

12.5. Do I have access to my data collected and processed during the trial and can I rectify them? 25

12.6. Who, other than the Investigator and his staff, has access to my personal data? ... 25

12.7. What will happen to the results of the trial? 26
12.8. Will my data be used for other purposes than for the trial in which I take part? 26
12.9. How long will my data be kept? 26

13. Which biological samples are collected from me during the trial and what will happen with them? 27

13.1. Which biological samples are collected from me during the trial? 27
13.2. What will happen to the collected biological samples? 27
13.3. How will my biological samples be handled? 27
13.4. What happens with any remainders of biological samples once the analyses described in this document have been carried out? 27
13.5. Will any additional biological samples be collected and used for additional research? 28

14. Who has reviewed and approved the trial documents? 28
15. What happens in case of incidental findings? 28

CHAPTER II - INFORMED CONSENT 29
PARTICIPANT 29
LEGAL REPRESENTATIVE (REF.) 31
IMPARTIAL WITNESS / INTERPRETER (REF.) 32
INVESTIGATOR 33

GLOSSARY 34

REFERENCES 35

The trial at a glance

Dear patient,

You have a pancreatic adenocarcinoma that is potentially resectable (called borderline resectable). Currently, a surgical resection with free margins (= without leaving tumoural cells behind or too close to the resection margins) is the only treatment modality offering a chance of healing. Though at this stage of your health situation and tumor evaluation, an immediate resection of your pancreatic tumour is at risk of not meeting these conditions of free margins. Preliminary data from clinical studies have shown that treatments administered before surgery (what we call neoadjuvant treatments), reduces the tumour size and improves the surgery outcome and prognosis of potentially resectable pancreatic adenocarcinoma.
In this study, we want to demonstrate whether the addition of **stereotactic body radiation therapy** (**SBRT**: a type of radiation therapy that allows highly precise delivery of high doses to the tumour in a few sessions [5 sessions in this study]) to neoadjuvant chemotherapy might be of benefit to you. This benefit may result in an improved prognosis as well as a reduction of the size of the tumour and the contact(s) between your tumour and surrounding vessels, so that the tumour may be more likely to be curatively resected with free margins.

Therefore, I invite you to participate in this clinical trial called the "STEREOPAC Trial" to evaluate two different neoadjuvant treatments consisting of chemotherapy with or without SBRT, followed by surgery and adjuvant treatment (= treatment administered after surgery).

This study has 2 treatment arms: arm A and B.

Patients in arm A will receive a neoadjuvant treatment by chemotherapy alone followed by surgery.

Patient in arm B will receive a neoadjuvant treatment by chemotherapy in combination with SBRT followed by surgery.

After surgery, an adjuvant treatment with chemotherapy is indicated in both arms unless your condition precludes it.
The type of neoadjuvant treatment you receive is awarded to you by lottery, called randomization. You and your doctor know what treatment you are receiving.
Even if the treatments received in this study prove to be beneficial for you, a relapse or worsening of the disease may still be possible in the future.

In both arms, the neoadjuvant chemotherapy treatment delivered is called mFOLFIRINOX (= mFFX) and consists of a combination of several chemotherapeutic drugs: oxaliplatin + leucovorin or levofolic (= folinic acid) + irinotecan + 5-fluorouracil (5-FU) or capecitabine.
In case of intolerance to mFFX, another combination of chemotherapy called Gemcitabine-Nab-Paclitaxel (Gem + Nab-P) can be administered.

You will receive the chemotherapy medicines through an IV directly into a vein (this is what all patients in your situation receive). These medicines are standard and approved by the Belgian Authorities.

The stereotactic radiation therapy treatment (SBRT), that you will receive if you are allocated to the Arm B, is also a therapeutic option approved by the Belgian Authorities and was safely tested in preliminary clinical studies for potentially resectable pancreatic adenocarcinoma.

Before agreeing to participate in this study, we would like to provide you with more comprehensive information about its organizational, risk- and benefit implications. In this way you can decide for yourself whether you want to participate or not. This is called 'informed consent'.

This chapter already gives you an idea of what this study entails. Nevertheless, we would like to ask you to read all pages, even if it takes some time. It is important that you read and understand everything. If you don't, you will participate in the study without knowing what you are committing to. If you have any questions, do not hesitate to ask me or the study staff.

If you agree to participate in the study, you will be required to undergo a number of medical examinations to verify that you meet all the requirements to be admitted to this study.

Once the treatment administration period has passed, you will remain in the study and will be followed by study personnel at the hospital to monitor your health.

All medicines and treatments have **side effects**. These side effects can sometimes even be serious. It is therefore very important that you **report** these side effects or new health problems **to me** as soon as they arise.
If this applies to you, you must not become pregnant or conceive another person while participating in the study and for a period after treatment has stopped. I will discuss effective methods of contraception with you. If you must use a method of contraception, it will not be reimbursed by the sponsor.

The sponsor, CUB Hôpital Erasme (ULB), has taken out an insurance for this study that reimburses the costs of the damages, in case this occurs, incurred directly or indirectly related to your participation in the study.

The Department of Gastroenterology, Hepato-Pancreatology and Digestive Oncology Hôpital Erasme (ULB) developed this study and asked me, along with other researchers and hospitals, to conduct it and reimburses us for this study.

All examinations and treatments that you undergo or receive as part of your participation in this study are either considered as standard of care and reimbursed by your health insurance or is free of charge for you if it is a part of the trial. Any other tests or treatments that you would have undergone or received if you had not taken part in the study must be paid for by your health insurance (mutuality) and by yourself.

The data collected during this research will be treated confidentially. Your doctor will pseudonymise your data (your identity will be replaced by an ID code in the study) before transfer to the sponsor. The latter will therefore not be able to identify you. Your biological samples will be pseudonymised in the same way.

This information and informed consent document may be linked to the information and informed consent document for the ancillary biological study vs 1.0 of June 27, 2022, in which you can optionally participate. This ancillary study is linked to this study and will also be proposed to you by your doctor.
For this ancillary study supplementary examination is performed on your residual tumor samples which are collected before and after treatment and on your blood samples collected before study start and taken during the study.

If you wish to participate in this biological ancillary research you must indicate this in the informed consent document for the ancillary biological study. It is important to think about it carefully. You may agree to participate in the main study and refuse to participate in this associated biological study.

One thing I would like to stress strongly is that you are under no obligation to participate in this study at all. Even if you have started the study, you can leave the study at any time. Of course, I and the medical team will continue to take care of you as before.

The Belgian authorities and an ethics committee ensure that your participation in this study does not harm you. Just because they approved this study doesn't mean you should feel compelled to participate.

In order to participate in this study, for your safety, you must agree that I will notify your treating physicians as investigator of your participation in this study. You should not participate in another clinical trial at the same time without informing the investigator or study personnel. We may refuse your participation for certain reasons. It is also very important that you cooperate and follow the instructions of the study staff and myself regarding the study.

If you agree to participate, you must sign the informed consent form. I will also sign this form and confirm that you have received the necessary information about this study. You will receive a dated and signed copy of the form.

Now that you already have an idea of what this research entails, feel free to read the other pages of this document. You don't have to do all of that at once. It is especially important to understand what you are reading. If you wish, you may also discuss this study with other trusted people (such as your doctor, family or friends). My colleagues and I are also ready to help you if there are things that are not clear. It is our duty to make sure you understand everything correctly.

Cordial greetings
Your treating physician and investigator

CHAPTER I – DESCRIPTION OF THE TRIAL AND YOUR RIGHTS WHEN PARTICIPATING

1. Why are we doing this trial

You are invited to participate in a clinical trial (hereinafter referred to as the "STEREOPAC Trial") to evaluate the safety and efficacy of the pre-operative administration of mFOLFIRINOX (mFFX) or Gemcitabine-Nab-Paclitaxel (Gem-Nab-P, in case of intolerance to mFFX) followed or not by stereotactic body radiotherapy (SBRT: a type of radiation therapy that allows highly precise delivery of high doses of radiation to the tumour in a few sessions [5 sessions in this study])) as treatment for your borderline resectable pancreatic adenocarcinoma (pancreatic cancer).
Surgical resection with free margins (=without leaving tumoural cells behind or close to the resection margins) is the only curative treatment for patients with pancreatic cancer. Unfortunately, most of these tumours are in direct contact and/or invading the major vessels around the pancreas and are at a locally advanced or borderline resectable stage, precluding a curative surgical procedure. Neoadjuvant treatment (preoperative treatment with chemotherapy and/or radiotherapy), has proven to make resection of the tumour possible and optimizes surgical outcome. However, the exact protocol of chemotherapy and radiotherapy remains to be determined for borderline resectable adenocarcinoma of the pancreas.
In recently reported trials, preoperative treatment with mFFX followed by radiotherapy, particularly SBRT, has shown promising benefit for patients in terms of surgery outcome and prolonged survival.

The aim of this study is to evaluate the safety and efficacy of preoperative treatment with mFFX with or without SBRT before surgery.
The preoperative chemotherapy treatment mFFX consists of several chemotherapeutic drugs: oxaliplatin + leucovorin or levofolic (= folinic acid) + irinotecan + 5-fluorouracil (5-FU) or capecitabine.
In case of intolerance to mFFX, Gem + Nab-P is administered.
After surgery adjuvant treatment is usually indicated unless your condition precludes it.

2. Why am I being asked to take part?

You are asked to participate in this study because you have a borderline resectable adenocarcinoma of the pancreas. This means the cancer is too close to the main vessels around the pancreas. Therefore, there is a high risk of undergoing a surgery without free margins (what we call a surgery with tumoral cells left behind or too close to the surgical margins). The borderline resectability of your tumour has been confirmed by a multidisciplinary medical board consisting of at least an oncologist, a gastroenterologist, a surgeon and a radiologist.

Preliminary data from clinical studies have shown that treatments administered <u>before surgery</u> (what we call <u>neoadjuvant treatments),</u> reduces the tumour size and improves the surgery outcome and prognosis of potentially resectable pancreatic adenocarcinoma. In your medical situation, it would be recommended that you receive preoperative chemotherapy using standard drugs. The exact benefit of adding stereotactic radiation therapy to preoperative chemotherapy is still not definitely proven, although promising.

Therefore, I invite you to participate in a clinical trial (called the "STEREOPAC Trial") to evaluate the safety and efficacy of chemotherapy with or without stereotactic radiotherapy before surgery.

The investigator or trial staff will discuss with you the requirements to be allowed to enter the trial.

You must be 18 or older and be in good general condition. Your blood parameters (red blood cells (hemoglobin), platelets, white blood cells, kidney and liver function) should also be normal or close to normal (acceptable values were defined for this study) to avoid adverse effects that could be induced by chemotherapy administration.

The tumor markers in your blood will also be assessed in order to monitor the evolution of your tumor.

If you are a woman of childbearing potential, you must use an effective method of contraception during treatment and at least one month before the first treatment and 6 months after the last administration of oxaliplatin, 5-FU (or capecitabine) and irinotecan (or Gem-Nab-P). If you are a male, an effective method of contraception must be used for the duration of treatment and at least 6 months after the last administration of oxaliplatin, 5-FU (or capecitabine) and irinotecan (or Gem-Nab-P).

You must of course agree to this before starting treatment.

You may not participate in this study:
- if you have another active cancer or an history of malignant pathologies in the past 5 years, with the exception of basal cell carcinoma of the skin, in situ cervical carcinoma or non-metastatic prostate cancer.
- If you have another concurrent anticancer therapy
- If surgery is contraindicated for your general condition or if you had a major surgery in the last 4 weeks
- if you have a history of radiotherapy of the upper abdomen
- if you have had prior treatment for pancreatic adenocarcinoma with at least 1 of the following chemotherapy treatments: oxaliplatin, irinotecan or 5-FU/capecitabine
- if you have infectious, cardiac or neurological uncontrolled pathologies.
- if you show a contraindication to the medicines used in this study.
- if you are pregnant or breast-feeding.
- if you are unable to consent to participate in this trial before starting treatment or if for medical or psychological reasons you cannot submit to the medical arrangements of the trial
- if you have a chronic concomitant treatment with the following strong inhibitors: cytochrome P450, family 3, subfamily a, polypeptide 4 gene (CYP3A4).
- if you are simultaneously participating in another clinical trial or if you are receiving or have received different treatment from another clinical trial in the 4 weeks prior to your participation in this clinical trial.

In addition to participating in the study, there may be other treatment alternatives available to treat your pancreatic cancer (e.g. treatments with other chemotherapy drugs). The investigator or a member of his team will discuss this with you.

You have pancreatic adenocarcinoma and your life-expectancy remains potentially limited and unpredictable whatever the therapy you receive. It is not guaranteed that your participation in this study will improve your quality of life or will extend your life.

3. Do I have to take part in a trial?

Your participation in a trial is voluntary and must remain free of any coercion. This means that you have the right not to take part in the trial or to withdraw at any time without giving a reason, even if you previously agreed to take part. Your decision will not affect your relationship with the investigator or your treating physician nor will it affect the quality of your future medical care.

If other treatments are available for your disease/condition, the investigator or his/her delegate will discuss these treatments with you. It could concern the following treatment:

Pretreatment with chemotherapy consisting of FOLFIRINOX (5-fluorouracil, irinotecan, oxaliplatin and leucovorin) or nab-paclitaxel combined with gemcitabine or radiotherapy, aiming to reduce the tumour invading the major vessels around the pancreas making resection of the tumour possible and optimizing surgical outcome.

4. What will happen during the trial?

This trial will include about 256 participants in Belgium at different hospitals participating in this study.

This study has 2 treatment arms, **arm A and B**.

Patients in **arm A** will receive neoadjuvant treatment by chemotherapy alone followed by surgery.

Patient in **arm B** will receive neoadjuvant chemotherapy in combination with radiation therapy (SBRT) followed by surgery.

The type of treatment you receive is awarded to you by lottery, called randomization.

This study is open, it means that the doctors and patients know which treatment is being administered.

Before any study related handling, your doctor will check whether you can participate in this study. He will make sure that your medical situation fulfils the inclusion criteria and that you do not present any contraindications to the treatment proposed.

<u>Pretreatment phase:</u>

During the screening visit you will receive all the information about the trial, you will be able to ask all your questions and will be asked to sign this informed consent if you accept to participate to this trial.

Once the informed consent signed, you will be asked to undergo some examinations to determine if you can participate to the trial:

- A physical examination including measures heart rate, respiratory rate, blood pressure, body temperature, height, weight
- An imaging evaluation assessing the progress of your disease by a CT scan of the chest, abdomen and pelvis, and an abdominal MRI with intravenous contrast fluid (IV). A Fluorodeoxyglucose (FDG)-positron emission tomography (PET)

scan can also be performed in addition to the CT scan and MRI previously described. For individuals who cannot undergo IV contrast CT, an MRI of the abdomen and pelvis with IV contrast fluid and a chest CT without contrast fluid is an acceptable alternative. An imaging assessment takes approximately 1 hour. There are no special precautions to be taken prior to this assessment.

- A blood assessment to evaluate your health condition (16 ml blood will be taken, it takes about 15 minutes to complete a blood draw): hemoglobin, platelets, white blood cells, kidney and liver function, total protein, glucose, chloride, sodium, potassium, calcium and magnesium, blood urea, lactate dehydrogenase and CRP (this is a protein associated with inflammation in the body).

  The blood tests are performed by your hospital laboratory. Your blood samples will be destroyed immediately after analysis. These blood tests would be performed in the same way as if you were not involved in this clinical study.

- Your tumour markers CA 19.9/CEA as a baseline to assess your disease evolution during treatment.
- A urine test to evaluate presence of blood or proteins in your urine (dipstick)
- An electrocardiogram to check your heart's electrical activity.
- Your doctor will also determine the activity of an enzyme present in your body (called DPD) that helps make your body resistant to the 5-fluorouracile infusion without generating significant side effects. This is not specific to the study, but is part of the standard examinations before starting treatment.

If you are a woman, you will also need to undergo a pregnancy test (which will be done at the time of the blood test) before you receive the first dose of chemotherapy.

During the pretreatment visit you will be asked to fill in quality of life scales as well as at some time points during the whole duration of the trial.

<u>Treatment phase:</u>

1. *<u>Chemotherapy cycles:</u>*

All included patients will receive 4 cycles of 2 weeks of mFFX chemotherapy.

In case of contraindication, intolerance or early non metastatic progression with mFFX, Gem + Nab-P regimen can be administered in 3 doses per 4 weeks.

Both mFFX and Gem + Nab-P are standardly used for pancreatic cancer.

These chemotherapy treatments are scheduled in the hospital every 2 weeks (or weekly depending on the drugs used). If you don't tolerate them well, they can be stopped or changed. Chemotherapy doses or dates when chemotherapy is given may be changed in case of serious adverse events.

Chemotherapy treatments can also be stopped if you decide to stop the treatment yourself or if your doctor makes this decision due to your medical condition.

The doctor who follows you will give you all the instructions regarding the course of chemotherapy treatment.

Continuous infusions are given with small infusion pumps, allowing you to remain independent. To enable chemotherapy infusions, a permanent venous access device (called an implantable chamber catheter or port-a-cath) is placed under the collarbone during a short hospital stay of less than 24 hours in a day hospital. This device is standard in the treatment of your disease and is also used outside the context of this study in the case of other chemotherapy treatments. The drugs can be easily injected into the device through the skin, eliminating the need to insert into a vein each time. The drugs enter the bloodstream directly. The device remains in place for the duration of the treatment, improves your comfort and facilitates intravenous drug administration.

When you arrive at the hospital for the chemotherapy, you will be installed in a chair or in a bed according to the hospital's habits.

First you will be administered oxaliplatin (85 mg / m2) intravenously over a 2-hour period. You will then be injected with leucovorin (400 mg / m^2; or 200 mg / m^2 levofolic) over a period of 2 hours. After leucovorin, irinotecan is injected at a rate of 165 mg / m^2 over a period of 90 minutes. A diffuser is then connected into your implantable chamber catheter. This diffuser contains a dose of 2000-2400 mg/m^2 of 5-FU which is diffused into your blood over 46 hours.

For the Gem+Nab-P regimen you will receive every week 2 x 30 minutes infusion of each drug (Nab-Paclitaxel 125 mg/m^2 and gemcitabine -1000 mg/m^2) during 3 weeks, followed by one week rest.

Before each administration of chemotherapy drugs during your treatment period, physical examination will be done and a blood assessment (16 ml of blood) will be performed to determine if your blood parameters allow you to continue your treatment or if adjustments in treatment doses are needed. Your blood values (hemoglobin, white blood cells and platelets), kidney and liver function, magnesium, total protein, glucose, sodium, potassium, chloride, serum calcium, blood urea, lactate dehydrogenase and CRP are assessed.

Just before the first administration of chemotherapy and, approximately 15 days after the last cycle of chemotherapy, 2 x 5 ml blood will be taken as well if you accept to participate to the optional ancillary biological study for which you may sign a separate informed consent form.

2. *Restaging and randomization*

At some timepoints during the study, the evolution of your disease under treatment is analysed by the tumour markers CA19.9 and CEA in the blood and by imaging. Imaging consists of a CT of the chest, abdomen and pelvis, an abdominal MRI and FDG-PET (optional). This is to evaluate if the pancreatic tumour has reacted posivitely on the chemotherapy +/- SBRT treatments. This is called restaging. If restaging shows progressive disease, you will be discontinued from the trial and your doctor will discuss further treatment with you (with the exception of an early non-metastatic progression under mFFX were a shift to Gem+Nab-P is authorized).

If the first restaging shows no progressive disease, you will be randomized (treatment will be awarded to you by lottery) to arm A or arm B.

If you are randomized to arm A, you will receive 4 extra cycles of mFFX (or 2 cycles (6 doses) of Gem + Nab-P) chemotherapy, followed by a CT/MRI and FDG-PET

(optional) evaluation to assess if the tumour is resectable. If this is the case you will undergo surgery.

If you are randomized to arm B, you will receive 2 extra cycles of mFFX (or 1 cycle (3 doses) of Gem + Nab-P) chemotherapy, followed by stereotactic radiotherapy and then followed by 1 or 2 additional cycles of chemotherapy of mFFX (or 1 cycle of Gem + Nab-P).

3. <u>Radiotherapy treatment is as follows (Arm B only):</u>

Before Isotoxic High Dose Stereotactic Body Radiation Therapy (iHD-SBRT), fiducial markers (small gold metal or polymer objects about the size of a grain of rice) are inserted into the pancreatic tumour through endoultrasonography route (flexible endoscopy under general anesthesia, usually performed in a one-day hospitalization) several days before the simulation of radiation therapy (in case of treatment with MR-Linac, the fiducial step can be skipped). MR-Linac is a new generation of linear accelerator (= the machine which delivers the radiotherapy treatment) because it is associated with an MRI and not with a CT scan. This allows for improved image quality during the radiation therapy treatment and the ability to perform daily adaptive treatments that can also improve the accuracy and the quality of pancreatic SBRT.

Fiducial markers are used as a benchmark during your treatment sessions, ensuring the high precision delivery of SBRT to the tumor and decreasing the rates of possible side effects.

They are totally inert and will stay in the pancreas until they are removed during the surgery with your tumour. In the event that surgery cannot take place for any reason, the fact that the fiducials remain in place in the pancreas does not pose a problem.

Before radiation treatments begin, you will go through a treatment planning process called "simulation." During this process, your radiation treatment team and doctor will perform a CT scan (and/or a MRI simulation) with intravenous contrast fluid (IV) and left a few marks on your skin where you will receive radiation. The marks will be given with a temporary paint marker and/or a small set (usually 3 points) of permanent tattoos. The position you are in for your simulation will be the same position you will be in every day for each session of treatment. Immobilization devices such as headrests, knee supports, or other devices may be used to help keep you in a comfortable position and make sure you don’t move during treatment.

Then, during 5 days (preferentially 5 consecutive days), the isotoxic high-dose (iHD)-SBRT will be delivered to you at the rate of one session per day. The iHD-SBRT is delivered to the pancreatic tumour with a simultaneous integrated boost, which allows to apply different radiation doses to different areas at the same time. The dose prescription of this SBRT is isotoxic, this means that <u>the dose delivered will be individually tailored for each patient</u> in order to allow:

- To not exceed the fixed predetermined levels of dose constraints and toxicity for the healthy organs (e.g., stomach, bowels…) located around the tumour in order to protect them as much as possible.
- To be able to individually escalate the dose delivered to the tumour and to the area of contact between the tumour and the major vessels to the highest authorized level in this study in order to maximise the expected beneficial effect of radiation therapy against the tumour.

Although it is personalized, the dose that you will receive will approximate the following dose regimen: 35Gy in 5 fractions with a simultaneous integrated boost to the tumour and the area where the major vessels are in contact with the tumour up to 55Gy.

In order to minimize the possible side effects related to SBRT, in addition to the isotoxic dose delivery mentioned above, your radiation oncologist will give you additional instructions to follow and will prescribe you in prevention some medications during the SBRT treatment. This iHD-SBRT will be explained to you in details by the radiation oncologist in charge of your therapy during a specific consultation.

4. *Surgery*

Surgery

If restaging after chemotherapy +/- radiotherapy shows that your pancreatic adenocarcinoma is operable, you will undergo surgery in one of the expert reference centres that participate to the trial. You will be invited to have a dedicated consultation with your surgeon at different times for discussing all questions you have concerning the surgery. Your investigator will discuss with you where the surgery will be performed.

Just before surgery you will be asked to again fill in the quality of life questionnaires.

At this phase of the study, if you have accepted to participate to the ancillary biological study for which you have signed a separate informed consent form, some residual tumoural tissue from the surgery will be taken for further analysis in the study..

Post-surgery

Approximatively 2 weeks after surgery you will undergo a physical examination, lab assessment, imaging and response evaluation in order to evaluate your medical situation.

At this phase of the study, 2 x 5 ml blood will be taken as well for further analysis in the study, if you accept to participate to the ancillary biological study for which you have signed a separate informed consent form.

Usually 8 weeks after surgery, only if sufficient recovery, you will be administrated adjuvant therapy for at least 4 months unless your medical condition precludes it.

After this visit, a follow up visit will be performed every 3 months for the 2 first years after surgery, then every 6 months for the next 3 years (5 years follow-up in total).

During these follow up visits you will be invited by the study team to undergo a physical examination, a blood assessment, an MRI/CT scan (+/- PET-CT) and you will be asked to fill in quality of life questionnaires (only every 6 months for the first two years).

Below is a detailed schedule of assessments:

	Before treatment	During treatment (chemotherapy and radiotherapy)	After treatment

		Before every chemotherapy dosing	15 days after Cycle 4	Restaging before surgery +/- 15 days after last treatment	FU 15 days after surgery	FU every 3 months during 2 years, then every 6 month during 3 years
Explanation about clinical and biological trial + Informed consent	X					
Clinical assessment (weight, height, general condition)	X	X	X	X	X	X
Blood test	X	X	X	X	X	X
Biological study additional blood samples	X			X		
Urine test	X					
Pregnancy test	X					
Electro Cardiogram	X					
CT scan, MRI, +/-FDG PET scan	X		X	X	X	X
Echo-endoscopy	X		X (only for SBRT arm)			
Quality of Life Questionnaires	X		X	X		X (at first FUP then every 6 months for the 2 first years)

Withdrawal of the study

It is possible that for different reasons, you will be withdrawn from the study.
After stopping chemotherapy because:

- Your illness is advanced or
- You become pregnant during treatment or
- You do not tolerate the treatment well (even after a shift to Gem+Nab-P) or
- Your treatment had to be suspended for 28 days or more or
- Intake of non-permitted medication or procedure or

- Insufficient patient compliance or
- Your doctor thinks it is better for you or
- You decide to withdraw your consent to participate in the study.

In this case, you will have to end your study participation and a follow-up consultation will be planned ± 15 days after stopping treatment to investigate possible side effects of the treatment by:

  - a full lab assessment + tumor markers
  - a physical examination
  - an assessment of your disease by a CT scan.

After this, a hospital consultation will be scheduled to find out how you are doing every 3 months for the 2 first years after surgery, then every 6 months for the next 3 years.

5. Will I benefit from the trial?

The information obtained during this clinical study will contribute to a better understanding of the use and the role of neoadjuvant treatments by chemotherapy and stereotactic radiotherapy in borderline pancreatic cancer, as well as to the potential future development of new therapeutic options through a better understanding of the tumour biology for your treatment or that of future patients (ancillary biological study).

In several recently reported preliminary trials, preoperative treatment with mFFX followed by radiation therapy, particularly SBRT, showed promising benefits for patients in terms of surgical outcomes (better resection rates, including with free margins) and prolonged survival.

The neoadjuvant treatment with chemotherapy +/- stereotactic radiotherapy applied in this study may or may not be beneficial for the treatment of the potentially resectable pancreatic cancer you have and/or reduce your symptoms. Even if it proves beneficial for you, recurrence/progression of the disease or worsening of symptoms is always possible.

6. What are the possible risks and discomforts of taking part?

6.1. What are the possible side effects of the treatment?

All medications can have known or unpredictable side effects. In view of the benefit-risk balance, previous studies have shown that the side effects of oxaliplatin, irinotecan, 5-FU, gemcitabine, Nab-paclitaxel and isotoxic high-dose stereotactic radiotherapy (iHD-SBRT) were acceptable.

These treatments are standard for your disease. Therefore, your risk for these side effects is the same as if you were not taking part in the study. Your doctor will monitor your health closely and when needed adjust the administered dose in a timely manner to minimize the risk of serious adverse effects.

However, you must be aware that you may experience the following side effects:

For oxaliplatin, the most common toxicities (≥ 1/10) are:

- ✓ Decrease in white blood cells, red blood cells (anemia), platelets (thrombocytopenia) in the blood
- ✓ Problems with the sensitivity of the fingertips and toes (peripheral sensory neuropathy, sensory disturbances),
- ✓ Change in taste, loss of appetite (anorexia)
- ✓ Skin disorders, hair loss, injection site reaction and allergy / allergic reactions
- ✓ Headache
- ✓ Digestive effects: nausea, diarrhea (soft or liquid stools, and frequent), vomiting, constipation
- ✓ Stomatitis (inflammation of the mouth), mucositis (type of mouth ulcers),
- ✓ Liver disorders (elevated liver enzymes, elevated alkaline phosphatase, bilirubinaemia and LDH),
- ✓ Decrease in blood ions (magnesium, potassium, sodium)
- ✓ Increase in blood glucose (hyperglycaemia)
- ✓ Difficulty breathing, coughing
- ✓ Nose bleeds
- ✓ Abdominal and back pain
- ✓ Fever
- ✓ Infections,
- ✓ Fatigue, asthenia (this is general body weakness)

<u>For 5-FU (5-fluorouracil), the most common toxicities (≥ 1/10) are:</u>

- ✓ Stomatitis, mucositis, diarrhea, anorexia, nausea, vomiting
- ✓ Decrease in white blood cells and platelets
- ✓ Infections
- ✓ Hyperuricaemia (too high a concentration of uric acid in the bloodstream)
- ✓ Reversible hair loss
- ✓ ECG disorders
- ✓ Slow scarring
- ✓ Nose bleeds
- ✓ Discomfort
- ✓ Fatigue, generalized body weakness

<u>For irinotecan, the most common toxicities (≥ 1/10) are:</u>

- ✓ Decrease in white blood cells, red blood cells (anemia), platelets (thrombocytopenia) in the blood
- ✓ Diarrhea
- ✓ Nausea, vomiting, weight loss, anorexia (loss of appetite)
- ✓ Stomach or bowel pain
- ✓ Stomatitis (mouth ulcers)
- ✓ Cholinergic syndrome (nervous system disorder)
- ✓ Dehydration
- ✓ Low salt levels in the blood
- ✓ Reversible hair loss
- ✓ Fatigue, generalized body weakness
- ✓ A dizzying feeling
- ✓ Fever

In case of intolerance to FFX, gemcitabine-nab-paclitaxel regimen can be chosen.

For gemcitabine, the most common toxicities (≥ 1/10) are:

- ✓ Decrease in white blood cells, red blood cells (anemia), platelets (thrombocytopenia) in the blood, bone marrow suppression
- ✓ Dyspnea (shortness of breath)
- ✓ Nausea and vomiting, diarrhea, weight loss, anorexia
- ✓ Hematuria (presence of blood in the urine)
- ✓ Reversible hair loss
- ✓ Muscle pain
- ✓ Cold shivers
- ✓ Fatigue, generalized body weakness
- ✓ Headaches
- ✓ A dizzying feeling
- ✓ Cough, running nose, transpiration
- ✓ Sleeping disorders
- ✓ Swelling and water retention in soft tissues (peripheral edema)
- ✓ Fever
- ✓ Severe hypersensitivity/allergic reaction with severe rash and red itchy skin
- ✓ Liver problems: detected by abnormal results of blood tests
- ✓ Proteins in urine

For Nab-P the most common toxicities (≥ 1/10) are:

- ✓ Decrease in white blood cells, red blood cells (anemia), platelets (thrombocytopenia) in the blood, bone marrow suppression
- ✓ Effect on peripheral nerves (pain, numbness, tingling or loss of sensation)
- ✓ Nausea, vomiting, diarrhoea, constipation, sore mouth, loss of appetite
- ✓ Weakness and fatigue, fever
- ✓ Reversible hair loss
- ✓ Swelling of mucous membranes and soft tissues
- ✓ Rash
- ✓ Pain in the joints
- ✓ Pain in the muscles
- ✓ Dehydration (dehydration), taste disorder, weight loss
- ✓ Low potassium levels in the blood
- ✓ Depression, sleeping troubles
- ✓ Headache
- ✓ Cold chills
- ✓ Respiratory difficulties, cough
- ✓ Dizziness
- ✓ Increases in liver function tests
- ✓ Abdominal pain
- ✓ Nosebleeds

For Isotoxic high-dose SBRT, the common toxicities (≥ 1/10) are:

- ✓ Fatigue
- ✓ Loss of appetite
- ✓ Difficult digestion
- ✓ Nausea, vomiting (an anti-nauseous drug will be prescribed to you by your radiation-oncologist in prevention during the 5 sessions of SBRT)
- ✓ Abdominal discomfort and pain

- ✓ Diarrhea, intestinal cramps
- ✓ Gas / bloating
- ✓ Temporary changes in blood work (decrease in blood counts, increase in liver enzymes) without symptoms
- ✓ Gastric,duodenal ulcer (without symptoms associated in around 50% of the cases; specific anti acid drugs will be prescribed in prevention of this risk)

Most side effects disappear a few weeks after the last radiation treatment.

It is very important that you report any new or worsening health problem to the investigator immediately. This is true even if you think it has nothing to do with the study, and even if it has already been described in this document. If you need to take other medications, discuss this with the investigator before taking them. If for any reason you consult another doctor during the study, you must inform him / her that you are participating in a study. This can be important for proper diagnosis and treatment if necessary.

6.2. What are the possible risks or discomforts of the examinations during the trial?

The clinical exams you will need to undergo are those that are usually performed routinely in this type of disease that requires chemotherapy (oral or intravenous).

All examinations (blood tests, scans, tumour biopsies to confirm diagnosis) are part of the standard examinations performed as part of consecutive assessments of pancreatic cancer.

Hospital admissions for chemotherapy administration and for implantation of the chamber catheter are also routine in this type of disease.

The implantable chamber catheter is placed under local anesthesia. This can cause local discomfort for a few days and is associated with a slightly increased risk of blood clots.

The tumour biopsy through echo-endoscopy that led to the diagnosis was taken before you entered this study. No new biopsy will be taken as part of this study.

The placement of fiducials through echo-endoscopy before the SBRT treatment in Arm B is also a standard procedure (the cost of the fiducials, currently not reimbursed, will be paid by the study sponsor).

Echo-endoscopies can cause transient inflammation of the pancreas or migration of biliary prosthesis if you have one.

Scan imaging occasionally causes an allergic reaction to the injection product (iodine allergy). These reactions can be prevented with premedication that will be prescribed by your doctor. If the allergic reaction persists or is too severe, another imaging modality can be used.

Drawing blood (approximately 16 ml of blood) needed for the routine analysis may cause pain, bleeding, bruising or local inflammation at the injection site. Some participants may also feel dizzy or even faint during the administration. The personnel performing the blood collection will make every effort to minimize these inconveniences.

6.3. Can I take other medicines during the trial?

Do not hesitate to ask your investigator for more explanation about the use of other medicines and food supplements.

6.4. Will my participation to the trial have an impact on my daily activities?

You are strongly advised not to use alcohol and tobacco during chemotherapy and radiotherapy treatment. Also, you will be asked not to eat grapefruit or drink grapefruit juice as this can increase the side effects of the chemotherapy. Temporary diet advices will be provided to you by your radiation oncologist (Arm B only) in order to diminish intestinal gas and bloating than can interfere with the proper course of the radiotherapy treatment.

If you experience drowsiness, dizziness or fatigue you will be asked not to drive, use machines, or perform other tasks that require your full attention.

6.5. Can my partner or I get pregnant or can I breastfeed during the trial?

This section is intended solely for participants with a potential to get pregnant or participants who may get their partners pregnant.

Female participant**:** Because the effects of chemotherapy medicines and radiotherapy on an unborn child or infant are not known, you will not be allowed to take part in this trial if

- you are pregnant,
- wish to become pregnant in the near future or
- if you are breastfeeding.

It is also not allowed to do egg/ovum donation during and after your participation in the trial for up to 4 months after the last intake of oxaliplatin, for up to 6 months after last intake of 5-FU and radiotherapy and for up to 1 month after last irinotecan intake.

If you take part in the trial, and if you are a woman of childbearing potential you must use an effective method of contraception during treatment and at least 6 months after the last chemotherapy administration or radiotherapy treatment. If you are concerned, please discuss this with your researcher. Please inform the investigator in case you would decide during the trial to change your method of contraception.

At the start of the study, before the first dose of study medication, you will need to have a pregnancy test (this will be taken along with your blood sample). A repeated pregnancy test must be done if you miss any periods or your menstrual cycle becomes irregular.

Nevertheless, if you become pregnant during the trial, you should inform immediately the investigator and your treating physician.

Male participant:

If you take part in the trial, you must use contraception and you should not be sperm donor for the duration of the trial and up to until 6 months after the last chemotherapy administration or radiotherapy treatment. Please discuss this point with the investigator if this applies to you.

You commit to inform your female partner of your participation in this trial and of the potential risk to an unborn child.

Nevertheless, if your partner becomes pregnant during the trial, you should inform immediately the investigator. If you agree, (s)he will contact your partner to ask her to be followed up during her pregnancy and its outcome and to sign a specific informed consent (for the pregnant partner).

7. What If something goes wrong within the trial?

Even if there is no fault, the sponsor is liable for harm caused to you whether directly or indirectly related to your participation in the trial. The sponsor has taken an appropriate insurance (a so called “No Fault insurance”) for this liability (Ref. 1). The insurer is Ethias Assurance. The policy number is 45.415.046. They can be reached at number 04/220.31.11 and speak French, Dutch and English. This way you can express yourself in one of these three languages. A copy of the insurance certificate can be obtained from the investigator or trial staff.

If you (or in the event of death, your rightful claimants) seek compensation for a harm to your health as a direct or indirect result of participating in the trial, you must inform your investigator or trial staff promptly.

If the investigator believes that a link between the new or worsened health problem(s) and the trial is possible, he/she will inform the trial sponsor. The sponsor will then immediately initiate the declaration procedure to its insurance company. If the company considers it necessary, it will appoint an expert to assess whether there is a link between your reported health problem(s) and the trial. The insurance does not cover the natural progression of your disease/condition or the known side effects of the treatment you would have received without taking part to the trial (*that is* your standard treatment).

Whenever you feel it is appropriate or if you or your rightful claimants disagree either with the investigator or with the expert appointed by the insurance company, you may contact the insurance company or proceedings may be brought against the insurance company. You will find the contact details on the front page of this form.

8. What if other treatment options or new information on the IMP become available during the course of the trial?

During the course of the trial, important new information might become available, possibly affecting your decision to (further) participate. For example, other treatments for your pancreatic cancer or important new information on the study treatment regimen may become available. It is the duty of the investigator to discuss this new information with you and to give you the opportunity to re-consider your participation in the trial.

If you decide to stop taking part in the trial or if you are no longer able to participate, your investigator will see to it that you continue to receive the best possible medical care.

9. Can my participation in the trial end prematurely?

As explained in detail below, your trial participation may end prematurely when

- you decide to withdraw your consent,
- the investigator decides to end your trial participation, or
- other entities interrupt or end the trial.

In any case, if your trial participation ends prematurely, the investigator will discuss your future medical care with you. The sponsor can continue to retain and use any data that have already been collected before the end of your participation. This is to avoid skewing / biasing results of the trial (as described in § 12.4.).

If you experience a side effect at the moment of stopping the study treatment regimen, the investigator may contact you in the future to see if it has resolved or not after the end of the trial participation.

If you experience a new side effect after the end of your trial participation you may contact the investigator to ask for a follow-up.

9.1. You decide to withdraw your consent

You are entitled to withdraw your consent for any reason, at any time, without having to justify your decision. However, for your safety, you should inform the investigator of your decision. Although it is not mandatory, it may be useful for the investigator and for the sponsor to know the reason of your decision (for example side effects, frequency of clinical visits).

If you withdraw your consent, this means you decide to stop

- the treatment with the IMP, and
- all trial-related visits and examinations.

Please discuss with your investigator to evaluate the practical modalities of your withdrawal (in light of your situation), including any follow up-visits or procedures.

In any case, no new data will be sent to the sponsor.

If your biological samples (e.g. blood samples, urine samples, residual tumour samples) have already been used or analysed before the withdrawal of your consent, the sponsor still has the right to use the results from those tests.

The biological samples that have been collected (but not tested) before the withdrawal of your consent and the data obtained from it, can also still be used by the sponsor. You may ask for a destruction of those samples. If this impacts the validity of the trial, the destruction may be postponed till the end of the trial.

In case you have signed an additional consent form for the use of your samples in future research, and you choose not to withdraw this separate consent, your samples can still be used for this research.

9.2. The investigator decides to end your trial participation

The investigator may end your trial participation because

- you become pregnant during the trial,
- it is better for your health,

- he/she determines that you are not following the instructions given to participants, or
- any other reason that will be explained.

9.3. Other entities may interrupt or end the trial

The sponsor and the competent Belgian health authorities may interrupt or end the trial because

- the information gathered shows that the study treatment is not effective (does not deliver a sufficient level of improvement in the health of the trial participants),
- the study treatment causes more (serious) side effects than anticipated, or
- any other reason that will be duly motivated by such party.

10. Which treatment will I get after my participation in the trial?

After you stopped the studied treatment, the investigator will assess your health. If necessary, he/she will prescribe you the best standard treatment available or refer you to another treating physician of your choice.

Several treatment options are available to you. These treatment options can be chemotherapy drugs, radiotherapy or surgery. These different treatment options will be discussed by your doctors who will determine the best treatment option for you based on your age, condition, co-morbidities (presence of one (or other) health problems related to your pancreatic cancer) and previous treatments you have received.

11. Will my participation in the trial involve extra costs for me?

11.1. Examinations and treatments paid by the sponsor

The sponsor compensates the hospital or site for

- the time devoted to the trial by the investigator and the trial staff,
- the visits/consultations and all scheduled examinations specific to the trial,
- material used for this study and not reimbursed (fiducials).

If you need more details or if you are not affiliated with a mutual insurance fund (Belgian social security), please contact the trial staff.

11.2. Other expenses

You have no additional research costs if you participate in this clinical study.

The treatments and evaluations of treatments efficacy (restaging) you will received in this study are considered as standard of care performed in an academic trial. This includes: chemotherapy, radiotherapy, surgery, imaging, blood draws [including if you are female, the additional pregnancy test in case you do not have your period or your menstrual cycle becomes irregular] and consultations. Therefore, these interventions

will be charged to your insurance fund and mutuality or to you if the amounts are not covered by these. This is all part of the standard care that you receive even if you are not participating in the study.

If you need more details or if you are not affiliated with a mutual insurance fund (Belgian social security), please contact the trial staff.

There is no additional reimbursement for transport (standard intervention depending on your mutual insurance fund), incapacity for work and contraceptives.

You will not receive any financial compensation for your participation in this study.

12. Which data are collected about me during the trial and what will happen with them?

12.1. Which data are collected and processed during the trial?

The collected and processed personal data concern information about your health and medical condition. This includes your medical history, some of your background information (for example your age, sex, and ethnic origin) and the results of examinations required by the trial.

12.2. How will the investigator treat my personal data?

The investigator is bound by professional secrecy about the data collected.

This means that he/she will never reveal your identity, including in a scientific publication or a lecture and that he/she will encode your data (*that is* by replacing your identity by an identification code in the trial) before sending them to the sponsor.

Therefore, the investigator and the trial staff under the responsibility of the investigator, will be the only ones able to establish a link between your identity and the data transmitted during the trial[1], with the exceptions listed under §12.6.

The data transmitted to the sponsor will not allow the sponsor to identify you[2].

12.3. What will happen to information about me collected during the trial?

Your participation in the trial means that your personal data

- are collected by the investigator, and
- are used in an encoded form by the trial sponsor.

The investigator and the sponsor can only use the encoded personal data for research purposes in connection with scientific publications within the context of the trial that you participate in, or for a broader use of the encoded data if described below.

[1] For clinical trials, the law requires this link with your records to be retained for 25 years. In the case of an advanced therapy medicinal product using human biological material, this period will be a minimum of 30 years and a maximum of 50 years in accordance with the Belgian Law of 19 December 2008 on the use of human biological material and the applicable royal decrees.

[2] The database containing the results of the study will therefore not contain any combination of elements such as your initials, your gender and your full date of birth (dd/mm/yyyy).

If wider use of the encoded data is planned, it will be noted below.

In addition, the sponsor may provide access to the encoded data to external researchers (that are not involved in this trial). In the event an external researcher wants to use the data in a project not yet described in this document, this project will have to be approved by an Ethics Committee. If your encoded trial data are sold, you will not benefit from this.

12.4. How will my data be handled?

Your trial data will be processed in accordance with the General Data Protection Regulation (GDPR, Ref. 2) and the Belgian law on data protection of 30th July 2018 (Ref. 3). The sponsor is responsible for this processing.

Processing your personal data in this trial is allowed because we are conducting scientific research and you have given your **consent.**

12.5. Do I have access to my data collected and processed during the trial and can I rectify them?

You are entitled to ask the investigator what data are being collected about you and how those data will be used in connection with the trial.

You have the right:

- to inspect and access these data
- to ask for correction if they are incorrect
- to withdraw your consent for the processing of personal data. However personal data collected before withdrawal will be kept to avoid skewing of results in the trial.

12.6. Who, other than the Investigator and his staff, has access to my personal data?

To verify the quality of the trial, it is possible that your personal uncoded data or information in your medical records relevant for the trial, will be examined by people outside the trial staff under the responsibility of the investigator. This access takes place under the supervision of the investigator and these persons are bound to professional secrecy or a confidentiality agreement. The following might be considered:

- the personnel designated by the sponsor of the trial (MONITORS and AUDITORS), and people or organisations providing services for or collaborating with the sponsor. They will however never transfer your name and contact details to the sponsor.
- inspectors of competent health authorities worldwide
- an independent audit group
- people designated by the Ethics Committee

For the needs of the trial, the encoded trial data may be sent to other EU and non-EU countries and may be reviewed by

- personnel (other than the inspectors) of competent health authorities of Belgium (Federal agency for medicines and health products, FAMHP) and other EU and non-EU countries,
- the evaluating Belgian Ethics Committee(s),
- external researchers,
- the sponsor of the trial, personnel designated by the sponsor, and people or organisations providing services for or collaborating with the sponsor, and/or
- group companies of the sponsor in Belgium, and in other EU and non-EU countries.

The European regulation and the Belgian legislation on data protection have requirements for transferring data to non-EU countries. The sponsor must ensure equivalent guarantees regarding personal data protection standards before transferring the encoded data to non-EU countries. If for this purpose, there is a data protection agreement, a copy of this agreement may be obtained via the investigator. You can always contact your investigator to obtain more information about any such transfers.

12.7. What will happen to the results of the trial?

After trial closure, a description and the results of this clinical trial will be published in specialised medical journals. A copy of the scientific publication or a summary that can be understood by a participant can be obtained from the investigator or the trial staff.

A description of the trial will also be available on https://www.clinicaltrialsregister.eu/ and/or https://www.Clinicaltrials.gov. You can search these websites at any time using the trial number given on the front page of the informed consent form. The websites will include a summary of the results within 1 year after the end of the trial (Ref. 4).

These websites or publications will not include information that can identify you.

12.8. Will my data be used for other purposes than for the trial in which I take part?

The results of the trial will be used to answer the scientific questions of the trial. In addition, the sponsor would like to use your data obtained from this trial, in connection with other research and development activities (and the associated scientific publications). These activities may concern the disease/condition for which the investigated medications are evaluated in this trial

Any additional research outside of the trial, must be approved by a Belgian recognized Ethics Committee.

You may agree or disagree to the use of your trial data for other purposes by ticking the appropriate check-box in Chapter II, page 30.

12.9. How long will my data be kept?

After the end of the trial your encoded data will be retained for at least 30 years (Ref. 5) to ensure the validity of the research. This will also be the case if you stopped trial participation prematurely.

13. Which biological samples are collected from me during the trial and what will happen with them?

13.1. Which biological samples are collected from me during the trial?

Biological samples are samples of human body material (for example blood, tissue, urine, faecal stool, etc.). These biological samples are taken as part of the blood and urine assessment taken prior to study treatment to see if you can participate in this study and during study treatment to determine if your blood parameters allow for your treatment or if treatment dose adjustments are needed.

13.2. What will happen to the collected biological samples?

Biological samples (taken as part of the blood and urine control before study treatment and during study treatment) are analysed at your hospital laboratory. They will be destroyed immediately after analysis. These biological samples are analysed for the the objectives of this trial.

The results of the analysis of your biological samples may by chance (and in addition to the objectives of the study) reveal information that may be important for your health or that of your blood relatives. These data are called "accidental finds". In this study, some biological samples will be anonymised. Anonymization means that your biological samples and your personal data can no longer be linked to your identity.

Therefore, these accidental finds cannot always be treated as described in Chapter I, § 15, and you cannot be informed of the analysis results.

If you do not agree with this anonymization, you cannot participate in this study.

13.3. How will my biological samples be handled?

The procedure to encode your biological samples is the same as that used for your personal data (see I § 12.3, page 24). Samples sent to the sponsor or to organisations working in collaboration with the sponsor, will only be labelled with your trial identification code.

As part of the trial, the sponsor might transfer (a part of) your samples to a laboratory that is working with them. This laboratory may only use your samples as specified in this document. The tracking of your samples will be ensured by the sponsor unless you have accepted anonymization of your samples.

Your biological samples are deemed to be a “donation”. You will not receive any financial benefit associated with the development of new therapies derived from the use of your biological samples, and which may have commercial value.

13.4. What happens with any remainders of biological samples once the analyses described in this document have been carried out?

The sponsor shall use the biological samples within the context of the trial as described above.

Your biological samples will be destroyed once the analyses for this trial have been carried out. You may also ask for a return of the remainders of your biological samples, if they could still be usable for your benefit. Please contact your investigator or the trial staff for this.

13.5. Will any additional biological samples be collected and used for additional research?

With your consent, the sponsor would also like to invite you to take part in additional research intended to gain a better understanding of the disease and its treatment. Your participation in this additional research is optional and will involve donating additional biological samples and some residual tumour tissue (if available).

We will provide you with more information specific to this research in a separate informed consent form. If you want to participate in this additional research we ask you to sign this separate informed consent form.

14. Who has reviewed and approved the trial documents?

The documents of the trial have been reviewed by:

- The Belgian competent health authorities (FAMHP) or if applicable by the competent national health authorities of other EU members states and
- An independent Belgian Ethics Committee

It is the task of the competent health authorities and the Ethics Committees to protect people who take part in a trial. The health authorities will ensure that the trial is conducted in accordance with the applicable legislation.

You should not under any circumstances take their approval as an incentive to take part in the trial.

15. What happens in case of incidental findings?

If by chance and in addition to the trial objectives a result is discovered during the trial that may be important to your health or the health of your blood relatives (called ”incidental findings”), the sponsor will inform the investigator. With your consent the investigator will notify you and your treating physician about your results and potential consequences. If necessary, the investigator and/or the treating physician will advise you on the next steps.

You agree or disagree to being informed of it by ticking the appropriate check-box in Chapter II,30.

CHAPTER II - INFORMED CONSENT

PARTICIPANT

PREREQUISITES FOR YOUR PARTICIPATION IN THE TRIAL

- I declare that I have been informed of and that I understand the purpose of the clinical trial, its duration, possible risks and discomforts, the precautions that I have to take and what is expected of me. My rights have been explained to me and I have understood those rights.
- I have had enough time to think about taking part in this trial and to discuss it with a trusted person (for example friends, relatives, treating physician, …).
- I have had the opportunity to ask any questions that came to mind and have obtained a satisfactory response to my questions.
- I understand that my participation in this trial is voluntarily and free from any coercion and that I am free to stop at any time my trial participation.
- I understand that data about me will be collected and that they will be treated confidentially.
- I agree to my personal data being processed as described in Chapter I, § 12, page 24.
- I understand that the sponsor has taken out an insurance in case I should suffer any damage in connection with my participation in this trial.
- I understand that when participating in this trial, I will not have any costs except those related to the standard of care treatment of my disease.
- I agree to my treating physician(s) being informed of my participation in this trial.
- I agree not to take part in any other trial at the same time without first informing the investigator or the trial staff, who might not permit me to participate for a good reason.
- I understand that I need to cooperate and follow the investigator's and trial staff's instructions regarding the trial.
- I understand that participation to the trial might end for me without my consent if I need other treatment, do not follow the trial plan, have a trial-related injury, or for any other justified reason.
- I am aware that the results of the analysis of any anonymized biologicals samples and the possible remainders of samples will not be available for me (Chapter I, § 13.2, page 27).
- I certify that all the information I have given about my medical history is correct. I understand that my failure to inform the investigator or designee about any exclusion criteria may harm myself.

OPTIONAL CONSENTS WHICH ARE NO PREREQUISITE FOR YOUR PARTICIPATION IN THIS TRIAL.

1. As specified in Chapter I, § 12.8, page 27, the sponsor would like to be able to use your data obtained from this trial in connection with other research and development activities (and the associated scientific publications) on the condition that such research purposes have been approved by a Belgian recognized Ethics Committee.

   Do you agree with the use of your data obtained in this trial for other research purposes?

   (Tick as appropriate. If you leave this question open, we assume the answer is 'I do not agree'.)

**☐ I agree**	**☐ I do not agree**

2. As described in Chapter I, § 13, page 27, and § 15, page 28, it may happen that incidental findings are discovered that may be important to your health or the health of your blood relatives.

   If this happens: do you want the investigator to inform you (directly or via your treating physician) of this result?

   (Tick as appropriate. If you leave this question open, we assume the answer is 'yes, I want to be informed'.)

**☐ No, I do not want to be informed**	**☐ Yes, I want to be informed**

I consent to take part in the trial, with the above restrictions and I have received a signed and dated copy of all pages of this document.

<u>Participant's surname and first name:</u>

<u>Date (DD/MMM/YYYY):</u>

<u>Participant's signature:</u>

Legal representative (Ref. 6)

I declare that I have been informed that I am being asked to take a decision on whether or not to take part in the clinical trial for the person I represent, considering his/her best interests and taking into consideration his/her likely wishes. My consent applies to all the items listed in the consent of the participant.

I have also been informed that as soon as the clinical situation allows, the person I represent will be made aware of his/her participation in a clinical trial and from that point will be free to continue with this participation or end it by signing or refusing to sign this consent form.

I have received a signed and dated copy of this document.

Legal representative’s surname and first name:

Relationship to the participant:

Date (DD/MMM/YYYY):

Legal representative’s signature:

IMPARTIAL WITNESS / INTERPRETER (REF. 7)

I, the undersigned (Tick as appropriate),

☐ Impartial Witness

☐ Interpreter

was present during the entire process of informing the participant and I confirm that the information on the objectives and procedures of the trial was adequately provided, that the participant (or his/her legal representative) apparently understood the trial and that consent to participate in the trial was freely given.

I declare furthermore that acting as an impartial witness, I am independent of the sponsor and the investigator.

Impartial Witness / Interpreter surname and first name:

Impartial Witness / Interpreter qualification:

Date (DD/MMM/YYYY):

Impartial Witness / Interpreter signature:

INVESTIGATOR

I, the undersigned investigator, confirm that

- the participant has been verbally provided with the necessary information about the trial, has been explained the content and has been given an original signed document.
- I have verified that the participant has understood the trial.
- I have given the participant sufficient time to agree to take part and to ask any questions.
- no pressure was applied to persuade the participant to agree to take part in the trial.
- I operate in accordance with the ethical principles set out in the latest version of the “Helsinki Declaration”, the “Good Clinical Practices” and the Belgian Law (Ref. 8).
-

<u>Investigator’s delegate, surname and first name:</u>

<u>Investigator’s delegate, qualification:</u>

<u>Date (DD/MMM/YYYY):</u>

<u>Investigator’s delegate signature:</u>

<u>Investigator’s, Surname and first name</u>:

<u>Date (DD/MMM/YYYY):</u>

<u>Investigator’s signature:</u>

GLOSSARY

FAMHP: Federal agency for medicines and health products

DPA: The Data Protection Authority ensures that personal data are handled with care and thoroughly protected, and that your future privacy also remains guaranteed.

NO FAULT INSURANCE:

The sponsor is liable for any injury or any damage that the participant has suffered, and which is directly or indirectly related to the clinical trial. You do not have to prove any mistake in this respect.

MONITOR and AUDITOR:

Both the monitor and auditor work for the sponsor.
The monitor takes care of a continuous quality check during the course of a trial. The auditor performs a quality check after the trial. They verify if the trial is being/was conducted according to the protocol, if the reported data are liable and if the clinical trial was conducted according the applicable rules.

REFERENCES

[1] This is in accordance with Article 29 of the Belgian Law of 7 May 2004 related to experiments on humans.

[2] General Data Protection Regulation No 2016/679 of the European Parliament and of the council of 27 April 2016 on the protection of natural persons with regard to the processing of personal data and on the free movement of such data, and repealing Directive 95/46/EC.

[3] The Belgian Law of 30 July 2018 on the protection of natural persons with regard to the processing of personal data.

[4] In accordance with section 4.3. of the Commission Guideline: Guidance on posting and publication of result-related information on clinical trials in relation to the implementation of Article 57(2) of Regulation (EC) No 726/2004 and Article 41(2) of Regulation (EC) No 1901/2006 - (2012/C 302/03). [From the moment the Clinical trial regulation enters into force : In accordance with article 37 of the Clinical trial regulation No 536/2014 of the European Parliament and of the council of 16 April 2014 on clinical trials on medicinal products for human use, and repealing Directive 2001/20/EC; sponsor have to provide summary results of clinical trials in a format understandable to laypersons.]

[5] In accordance with article 58 of the Clinical trial regulation No 536/2014 of the European Parliament and of the council of 16 April 2014 on clinical trials on medicinal products for human use, and repealing Directive 2001/20/EC.

[6] When a person of full age is incapable of expressing his will, legal representation must be used which is determined in successive order (administrator, or failing that, the spouse, the legal cohabiting partner, de facto cohabiting partner, an adult child, a parent, an adult brother or sister). The regulation is laid down in article 8 of the law of 7 May 2004 on experiments on the human person.

[7] Use of an impartial witness is necessary when either the subject or the subject's legally authorized representative speaks and/or fully understands the language of the approved informed consent form, but cannot read and write due to any physical impairment or is visually impaired. An interpreter is necessary when the investigator doesn't speak the language of the patient.

[8] Belgian Law of 7 May 2004 related to experiments on humans, and the applicable royal decrees.
