# Supplementary material for: Preoperative treatment with mFOLFIRINOX or Gemcitabine/Nab-paclitaxel +/- isotoxic high-dose stereotactic body Radiation Therapy (iHD-SBRT) for borderline resectable pancreatic adenocarcinoma (the STEREOPAC trial): study protocol for a randomised comparative multicenter phase II trial
Source: BMC Cancer. 2023 Sep 21;23:891. doi: 10.1186/s12885-023-11327-x (PMC10512504; doi:10.1186/s12885-023-11327-x)
Supplement: Supplementary file 3 — Supplementary Material 3 [file 12885_2023_11327_MOESM3_ESM.pdf]

Optional ancillary biological study (tumour and blood sample), associated with the main clinical study

STEREOPAC

Preoperative treatment with mFOLFIRINOX (or Gem-Nab-P) +/- isotoxic high-dose Stereotactic Body Radiation Therapy (iHD-SBRT) for borderline resectable pancreatic adenocarcinoma: a randomised comparative multicentre phase II study

Short title: STEREOPAC

EU number: 2022-501181-22-01

Sponsor: CUB Hôpital Erasme (ULB), Route de Lennik 808, B- 1070 Brussels

Coordinating center: CUB Hôpital Erasme (ULB), Route de Lennik 808, B-1070 Brussels

Coordinating Investigator for Belgium: Prof. Dr. JL Van Laethem, CUB Hôpital Erasme (ULB), Department of Gastroenterology, Hepato-Pancreatology and Digestive Oncology, jl.vanlaethem@erasme.ulb.ac.be, Phone: +32 2 555 3714

Ethics Committee that issued the opinion on the study: CTIS & FAGG submission

Participating centers:

Prof. Dr. J-L Van Laethem (CUB Hôpital Erasme (ULB), Route de Lennik 808, B- 1070 Brussels)
Prof. Dr. I. Borbath (Cliniques Universitaires Saint-Luc, Avenue Hippocrate 10, 1200 Brussels)
Prof. Dr. T. Vandamme (Universitair ziekenhuis Antwerpen, Wilrijkstraat 10, 2650 Edegem)
Prof. Dr. K. Geboes (UZ Gent, Corneel Heymans 10, 9000 Gent)
Dr. G Houbiers (Clinique CHC MontLégia, Bd Patience et Beaujonc 9, 4000 Luik)
Dr. P. Vergauwe (AZ Groeningen, President Kennedylaan 4, 8500 Kortrijk)
Dr Christelle Bouchart (Institut Jules Bordet, Rue Meylemeersch 90, 1070 Anderlecht)
Dr Francesco Puleo (CHIREC Boulevard du Triomphe 201, 1160 Brussels)
Dr. A. Dermine (Pôle Hospitalier Jolimont, Rue Ferrer 159, 7100 Haine Saint Paul)
Dr. S. Dingenen (Centre Hospitalier Universitaire et Psychiatrique de Mons Borinage (CHUPMB), Boulevard Kennedy 2, 7000 Mons)

Version No.	Version Date	Revision description
1.0	27 June 2022	Initial version

Who can I contact in case of questions?

Name	Function	In case of	Contact details
Surname, first name	Principal Investigator of the site	Information, problems or concerns	
	The trial staff	Information, problems or concerns	
	Emergency contact	Emergency	
	Patient rights ombudsman	Concerns relating to your rights as a participant in a trial	
Ethias Assurance<br>Rue des Croisiers, 24<br>4000 Liège	Insurance Company of the sponsor	In case of disagreement or complaint on a damage claim	Policy N°: 45.415.046<br>Phone N°: 04/220.31.11
	Data protection officer of the **site**	Questions relating to the confidentiality of your data	
	Belgian Data Protection Authority	Complaints relating to the confidentiality of your data	E-Mail: contact@apd-gba.be

PART I – DESCRIPTION OF THE TRIAL AND YOUR RIGHTS WHEN PARTICIPATING

Introduction

After consultation with your physician, you have decided to participate in the STEREOPAC main study and you will receive chemotherapy+/-stereotactic radiotherapy treatment. You received a separate information and consent form for this purpose.

As part of your participation in the main study, you are also invited to participate in an optional ancillary biological study (additional research of your blood and residual tumour sample), associated with the main study.

Before you agree to take part in this associated biological study, we invite you to take note of its implications in terms of organisation, possible risks and benefits, to allow you to make a decision with full awareness of the implications. This is known as giving “informed consent”.

This information and consent document must be combined with the information and consent document of the main study, version 1.0 of 27 June 2022.

Please read these few pages of information carefully and ask any questions you want to the investigator or his/her representative. There are 2 parts to this document: the information essential to your decision and your written consent.

If you take part in this ancillary biological study, you should be aware that:

- ➢ This clinical study is being conducted after having been reviewed by an independant Ethics Committee (CTIS & FAGG approval).
- ➢ Your participation is voluntary and must remain free from any coercion. It requires the signature of a document expressing your consent. Even after having signed this document, you can stop taking part by informing the investigator. Your decision not to take part or to stop taking part in the study will have no impact on the quality of your care or on your relationship with the treating physician(s).
- ➢ The data collected on this occasion are confidential and your anonymity is guaranteed during publication of the results.
- ➢ Insurance has been taken out in case you should suffer any damage in connection with your participation in this ancillary biological study.
- ➢ You will not incur any charges related to your participation in this ancillary biological study.
- ➢ You may contact the investigator or a member of his/her team at any time should you need any additional information.

Objectives

Currently, the influence of biological and molecular factors of pancreatic cancer on the evolution of the illness and the response to treatment are not well known. The determination of certain molecular and genetic characteristics of your illness, which can be detected directly in the tumour or the blood stream (circulating tumour DNA) are of significant scientific interest to better understand the sensitivity to drugs or other anticancer therapy and the prognosis for that type of cancer. In the future, these

results will eventually enable to propose a customised treatment to patients suffering from an adenocarcinoma of the pancreas.

The purpose of this biological study is to better understand the molecular and genetic factors and the tumoural microenvironment (= the direct environment around the tumoural cells such as the immune cells, small blood vessels…) which are likely to predict the safety and efficacy of preoperative treatment (chemotherapy with or without additional stereotactic radiotherapy) to treat pancreatic adenocarcinoma.
Researchers try to identify these factors in the tumour DNA/RNA present in the blood stream (plasma), in the DNA/RNA of the cells of your tumour and in the other cells populations present in the direct tumour microenvironment.

In the STEREOPAC study, for the treatment of your pancreatic adenocarcinoma you will receive preoperative treatment (neoadjuvant therapy) mFOLFIRINOX (oxaliplatin + leucovorin + irinotecan + 5-fluorouracil (5FU) (or gemcitabine + Nab + paclitaxel (Abraxane) in case of chemical intolerance of intolerance to mFOLFORINOX) with or without additional stereotactic radiotherapy before surgery.

<u>If you accept to take part in this associated biological study, the following biological samples will be collected and used</u>:

- A sample of your blood will be collected prior to the beginning of your treatment. This sample (2 tubes of blood of 5 mL) will be taken at the same time as the blood sample used for your medical assessment prior to the first cycle of chemotherapy.
- Another blood sample (2 tubes of blood of 5 mL) will be collected before surgery +/- 15 days after receiving your last dose of chemotherapy, also at the same time as the blood sample used for your medical assessment.

- Two samples of your tumour will be sent to the research laboratory for analysis:
    - A residual tumour sample (= the remaining tumoural tissue that is not used after the standard analysis required for the management of your pancreatic cancer) will be collected from the biopsies realised at the moment your illness was diagnosed before the start of the study treatment.
    - A second residual tumour sample will be collected from the surgical specimen (= the resected pancreatic tumour) issued of your surgery after you have received the neoadjuvant treatment with chemotherapy +/- stereotactic radiotherapy at the end of the study.

Your blood samples and your tumour samples described above and dedicated for research will be stored until the end of the trial at the laboratory of your hospital. At the end of the study, they will be sent to the biobank (= a bank that collects and stores biological samples for analysis) of CUB Hôpital Erasme. If you consent to this associated biological study, it will also mean that you agree to the storage of these biological samples for 30 years. If not all of the samples have been used for research purpose, they will be destroyed after 30 years.

The main purpose of this combined biological study in terms of your blood and residual tumoural samples will allow us to answer the following questions:
- Is the rate of circulating tumour DNA prior to the start of the treatment an actual prognostic indicator?*

- Are there prognostic and/or predictive factors that can be used on the biopsy samples (before any treatment) and that can help us to better guide the patient care? *
- Does the apparition of one or several resistance mutations to the studied chemotherapies and radiotherapy predict radiologic progression?
- Is there a way to easily identify distinct groups of patients according to the genomic and immune specificities of their tumour in order to be able to offer to them the most appropriate treatment? Can we identify new molecular or genomic targets that can improve or lead to new therapeutic proposals for pancreatic cancer?

* A prognostic factor assesses the spontaneous evolution of the illness, regardless of the effect of the treatment. A predictive factor helps to predict whether a patient's cancer will respond to a specific treatment.

These genetic and tumoural microenvironment analyses are optional and not a prerequisite for your participation to the trial. You agree or disagree to take part in these genetic analyses by ticking the appropriate check-box in Part II "Informed consent to the use of biological material (tumour samples and blood samples)" on page 9.

If you choose to participate to this ancillary biological study, you will be asked to sign the consent form, of which you will receive a copy.

If you decline to participate in this ancillary biological study, this will not affect your further medical care.

You are also entitled to withdraw your consent at any time regarding the use of the research data collected within this study, if the treatment of such research data is not limited to the scope of the present study.

<u>Risks and disadvantages</u>

Risks relating to assessment procedure specific to the study

Drawing blood can (on rare occasions) cause pain, bleeding, bruising or a local infection at the site of the blood sample. Similarly, some patients can suffer from light-headedness or even fainting during the procedure. The staff who take the blood will do all they can to minimize these discomforts.

<u>Anticipated results</u>

It is highly unlikely that the results of the biological study will become available in the near future. This is due to the fact that the clinical and research studies can take a long time and that samples and data from numerous patients must be collected before the results will be known.
New information may be obtained from the physician that treated you as part of the STEREOPAC main study.
It may happen by chance (and in addition to the trial objectives), that the results of the analysis of your biological samples reveal information that may be important to your health or the health of your blood relatives. These data are called "incidental findings". You can accept or refuse to be informed of these discoveries or that your doctor will be informed.

Voluntary participation and withdrawal from the study

Your participation in this associated ancillary biological research is optional, completely voluntary and must remain free of any coercion: this means that you have the right not to take part in the study or to withdraw without giving a reason, even if you previously agreed to take part. Your decision will not affect your relationship with the investigator or the quality of your future therapeutic care and will not influence your participation in the main clinical study.

You will have sufficient time to decide whether or not you want to participate. Before signing, do not hesitate to ask any questions you feel are appropriate. Take the time to discuss matters with a trusted person if you so wish.

If you withdraw your consent to take part in this associated ancillary study, no new data may be sent to the sponsor.

If your biological samples (blood and tumour samples) have already been used or analysed before the withdrawal of your consent, the sponsor still has the right to use the results from those tests.

The biological samples (blood and tumour samples) that have been collected (but not tested) before the withdrawal of your consent and the data obtained from it, can also still be used by the sponsor. You may ask for a destruction of those samples. If this impacts the validity of the trial, the destruction may be postponed till the end of the trial.

Please contact the investigator to discuss the practicalities of withdrawing from the study.

If you agree to participate, you will sign the informed consent form. The investigating will also sign this form and confirm that he or she has provided you with the necessary information about the study. You will receive the copy intended for you.

Guarantee of confidentiality and data protection

With respect to the protection of your privacy and therefore the protection of the analyses we will perform of your samples, your anonymity will be guaranteed by the measures that are taken to pseudonymize your samples. The procedure for pseudonymizing the biological samples is the same as the procedure for your medical data. The samples transmitted will therefore only mention your identification code in the study. During the entire clinical study, the physician-researcher and his team will be the only persons that can make a connection between the transmitted data and your medical file.

Since scientific progress in this area is constant, the sponsor would like to, with your consent retain the remainders of your biological samples for 30 years. The sponsor will use them for future research, outside the trial that you will participate in, to better understand the disease, its treatment and the responses to this treatment, and study medication. The retention of the remainders of your samples goes together with the retention of the accompanying encoded personal data.

You agree or disagree to the retention of the remainders of your biological samples (blood and tumour samples) for future research by ticking the appropriate check-box in Part II "Informed consent to the use of biological material (tumour samples and blood samples)" on page 9.

If you agree, any future research, additional to what is described above, may only be conducted according to the legislation on the use of human tissue material[1] and with the approval of a Belgian recognized Ethics Committee. As a general rule, you will be asked to sign an additional informed consent form in which the additional research is specified. You may withdraw your consent for future research at any moment.

Your trial data will be processed in accordance with the General Data Protection Regulation (GDPR, Ref. i) and the Belgian law on data protection of 30th July 2018 (Ref. ii). The sponsor is responsible for this processing.

Processing your personal data in this trial is allowed because we are conducting scientific research and you have given your **consent.**

To verify the quality of the associated biological study, it is possible that your medical records will be examined by persons subject to professional secrecy and designated by the ethics committee, the sponsor of the study, inspectors of competent health authorities worldwide or an independent audit body. In any event, this examination of your medical records may only take place under the responsibility of the investigator (or under the supervision of one of the collaborators designated by him/her). The (pseudonymized) study data will be able to be sent to Belgian or other regulatory authorities, to the relevant ethics committees, to other doctors and/or to organisations working in collaboration with the sponsor.

They will also be able to be sent to other sites of the sponsor in Belgium and in other countries (EU and non-EU) where the standards in terms of the protection of personal data may be different or less stringent. As explained above, the transmitted data are pseudonymized[2].

Your consent to take part in this ancillary biological study therefore also implies your consent to the use of your pseudonymized medical data for the purposes described in this information form and to their transmission to the aforementioned people and authorities.

If you have any questions relating to how your data are being processed, you may contact the investigator. The data protection officer in your hospital can be contacted as well.

The latter's contact details are as follows:
…………………………………………………………

Finally, if you have a complaint concerning the processing of your data, you can contact the Belgian supervisory authority who ensures that privacy is respected when personal data are processed.

The Belgian supervisory authority is called:
Data Protection Authority (DPA)
Drukpersstraat 35,
1000 Brussels
Tel. +32 2 274 48 00
e-mail: contact@apd-gba.be

---

[1] This is in accordance with Article 21 of the Belgian Law of 19 December 2008 on the acquisition and use of human body material with a view to medical application to humans or scientific research, and the applicable royal decrees.

[2] The sponsor then undertakes to respect the constraints of the European General Data Protection Regulation (GDPR) and the Belgian legislation on the protection of natural persons with regard to the processing of personal data.

Website: https://www.dataprotectionauthority.be

Contact

If you need further information, but also if you have problems or concerns, you can contact the investigator or a member of his/her research team. You can find the contact on page 2.

PART II - Informed consent to the use of biological material (tumour samples and blood samples)

Participant

PREREQUISITES FOR YOUR PARTICIPATION IN THE TRIAL

- I declare that I have been informed of and that I understand the purpose of the clinical trial, its duration, possible risks and discomforts, the precautions that I have to take and what is expected of me. My rights have been explained to me and I have understood those rights.
- I have had enough time to think about taking part in this trial and to discuss it with a trusted person (for example friends, relatives, treating physician, …).
- I have had the opportunity to ask any questions that came to mind and have obtained a satisfactory response to my questions.
- I understand that my participation in this trial is voluntarily and free from any coercion and that I am free to stop at any time my trial participation.
- I understand that data about me will be collected and that they will be treated confidentially.
- I agree to my personal data being processed as described in Part I, section “Guarantee of confidentiality” and page 6.
- I understand that the sponsor has taken out an insurance in case I should suffer any damage in connection with my participation in this trial.
- I understand that when participating in this trial, I will not have any costs except those related to the standard of care treatment of my disease
- I agree to my treating physician(s) being informed of my participation in this trial.
- I agree not to take part in any other trial at the same time without first informing the investigator or the trial staff, who might not permit me to participate for a good reason
- I understand that I need to cooperate and follow the investigator’s and trial staff’s instructions regarding the trial.
- I understand that participation to the trial might end for me without my consent if I need other treatment, do not follow the trial plan, have a trial-related injury, or for any other justified reason.
- I understand that genetic analysis will be conducted on my biological samples (blood and tumor samples).
- I certify that all the information I have given about my medical history is correct. I understand that my failure to inform the investigator or designee about any exclusion criteria may harm myself.

OPTIONAL CONSENTS WHICH ARE NO PREREQUISITE FOR YOUR PARTICIPATION IN THIS TRIAL.

1. As specified in Part I, section “Guarantee of confidentiality” page 6, the sponsor would like to be able to use your data obtained from this trial in connection with other research and development activities (and the associated scientific publications) on the condition that such research purposes have been approved by a Belgian recognized Ethics Committee.

   Do you agree with the use of your data obtained in this trial for other research purposes?

   (Tick as appropriate. If you leave this question open, we assume the answer is ‘I do not agree’.)

**☐ I agree**	**☐ I do not agree**

2. As specified in Part I, section “Objectives” on page 3, optionally, the sponsor will be conducting genetic analysis on your biological samples.

   Do you agree to the sponsor conducting genetic analysis on your biological samples?

   (Tick as appropriate. If you leave this question open, we assume the answer is ‘I do not agree’.)

**☐ I agree**	**☐ I do not agree**

3. As specified in Part I, section “Objectives” on page 4, the sponsor would like to retain the remainders of your biological samples for 30 years for future research outside the trial that you will participate in. The samples will be used to better understand the disease, its treatment and the responses to this treatment, and the study medication.

   Do you agree with the retention of the remainders of your biological samples and the accompanying personal data for future research outside the trial?

   (Tick as appropriate. If you leave this question open, we assume the answer is ‘I do not agree’.)

**☐ I agree**	**☐ I do not agree**

4. As described in Part I, section “Anticipated results” on page 5, it may happen that incidental findings are discovered that may be important to your health or the health of your blood relatives.

   If this happens: do you want the investigator to inform you (directly or via your treating physician) of this result?

   (Tick as appropriate. If you leave this question open, we assume the answer is ‘yes, I want to be informed’.)

**☐ No, I do not want to be informed**	**☐ Yes, I want to be informed**

I consent to take part in the trial, [if optional questions have to be answered by the participant: with the above restrictions and I have received a signed and dated copy of all pages of this document.

Participant’s surname and first name:

Date (DD/MMM/YYYY):

Participant’s signature:

LEGAL REPRESENATATIVE

I declare that I have been informed of the request to make a decision about participation in the study by the person I represent. I act in his / her best interest and take into account his or her possible wish. My consent applies to all points included in the participant consent form.

I have also been informed that as soon as the clinical situation permits, the person I represent will be notified of his / her participation in this study. At that time, he / she is free to consent to further participation or to discontinue participation by signing or not signing this consent form.

I have received a signed and dated copy of this document.

<u>Legal representative surname and first name</u>:

<u>Relationship with the participant:</u>

<u>Date (DD/MMM/YYYY):</u>

<u>Impartial Witness / Interpreter signature</u>:

IMPARTIAL WITNESS / INTERPRETER

I, the undersigned (Tick as appropriate),

☐ Impartial Witness

☐ Interpreter

was present during the entire process of informing the participant and I confirm that the information on the objectives and procedures of the trial was adequately provided, that the participant (or his/her legal representative) apparently understood the trial and that consent to participate in the trial was freely given.

I declare furthermore that acting as an impartial witness, I am independent of the sponsor and the investigator.

<u>Impartial Witness / Interpreter surname and first name</u>:

<u>Impartial Witness / Interpreter qualification</u>:

<u>Date (DD/MMM/YYYY):</u>

<u>Impartial Witness / Interpreter signature</u>:

INVESTIGATOR

I, the undersigned investigator, confirm that

- the participant has been verbally provided with the necessary information about the trial, has been explained the content and has been given an original signed document.
- I have verified that the participant has understood the trial.
- I have given the participant sufficient time to agree to take part and to ask any questions.
- no pressure was applied to persuade the participant to agree to take part in the trial.
- I operate in accordance with the ethical principles set out in the latest version of the “Helsinki Declaration”, the “Good Clinical Practices” and the Belgian Law [3].

Investigator’s delegate, surname and first name:

Investigator’s delegate, qualification:

Date (DD/MMM/YYYY):

Investigator’s delegate signature:

Investigator’s, Surname and first name:

Date (DD/MMM/YYYY):

Investigator’s signature:

---

[i] General Data Protection Regulation No 2016/679 of the European Parliament and of the council of 27 April 2016 on the protection of natural persons with regard to the processing of personal data and on the free movement of such data, and repealing Directive 95/46/EC.

[ii] The Belgian Law of 30 July 2018 on the protection of natural persons with regard to the processing of personal data.

---

[3] Belgian Law of 7 May 2004 related to experiments on humans, and the applicable royal decrees
